# A Comprehensive Survey of the Roles of Highly Disordered Proteins in Type 2 Diabetes

**DOI:** 10.3390/ijms18102010

**Published:** 2017-09-21

**Authors:** Zhihua Du, Vladimir N. Uversky

**Affiliations:** 1 Department of Molecular Medicine, Morsani College of Medicine, University of South Florida, 12901 Bruce B. Downs Blvd. MDC07, Tampa, FL 33620, USA; 2 Department of Computer Science, College of Computer Science and Software, Shenzhen University, Shenzhen 518060, China; 3 USF Health Byrd Alzheimer’s Research Institute, Morsani College of Medicine, University of South Florida, 12901 Bruce B. Downs Blvd. MDC07, Tampa, FL 33620, USA; 4 Laboratory of New Methods in Biology, Institute for Biological Instrumentation, Russian Academy of Sciences, Institutskaya str., 7, Pushchino, Moscow 142290, Russia

**Keywords:** type 2 diabetes mellitus, KEGG database, intrinsically disordered proteins, intrinsically disordered protein regions, protein–protein interaction, posttranslational modifications, disorder prediction

## Abstract

Type 2 diabetes mellitus (T2DM) is a chronic and progressive disease that is strongly associated with hyperglycemia (high blood sugar) related to either insulin resistance or insufficient insulin production. Among the various molecular events and players implicated in the manifestation and development of diabetes mellitus, proteins play several important roles. The Kyoto Encyclopedia of Genes and Genomes (KEGG) database has information on 34 human proteins experimentally shown to be related to the T2DM pathogenesis. It is known that many proteins associated with different human maladies are intrinsically disordered as a whole, or contain intrinsically disordered regions. The presented study shows that T2DM is not an exception to this rule, and many proteins known to be associated with pathogenesis of this malady are intrinsically disordered. The multiparametric bioinformatics analysis utilizing several computational tools for the intrinsic disorder characterization revealed that IRS1, IRS2, IRS4, MAFA, PDX1, ADIPO, PIK3R2, PIK3R5, SoCS1, and SoCS3 are expected to be highly disordered, whereas VDCC, SoCS2, SoCS4, JNK9, PRKCZ, PRKCE, insulin, GCK, JNK8, JNK10, PYK, INSR, TNF-α, MAPK3, and Kir6.2 are classified as moderately disordered proteins, and GLUT2, GLUT4, mTOR, SUR1, MAPK1, IKKA, PRKCD, PIK3CB, and PIK3CA are predicted as mostly ordered. More focused computational analyses and intensive literature mining were conducted for a set of highly disordered proteins related to T2DM. The resulting work represents a comprehensive survey describing the major biological functions of these proteins and functional roles of their intrinsically disordered regions, which are frequently engaged in protein–protein interactions, and contain sites of various posttranslational modifications (PTMs). It is also shown that intrinsic disorder-associated PTMs may play important roles in controlling the functions of these proteins. Consideration of the T2DM proteins from the perspective of intrinsic disorder provides useful information that can potentially lead to future experimental studies that may uncover latent and novel pathways associated with the disease.

## 1. Introduction

Diabetes mellitus (DM) is a metabolic disease characterized by hyperglycemia resulting from defects in insulin secretion, inefficiency of insulin action, or both. There are two major forms of DM: type 1 and type 2 diabetes, known as T1DM and T2DM, respectively. Although the loss of insulin activity causes an imbalance in glucose homeostasis and thereby leads to chronic hyperglycemia in both types, their pathogenic mechanisms are different. In fact, T1DM onset is associated with the autoimmune-mediated loss of the pancreatic insulin-secreting β-cells, whereas T2DM results from insulin resistance in target organs and defective β-cells leading to a shortage of hormone insulin [1]. According to the International Diabetes Federation (available online: www.idf.org), there are 415 million adults that have diabetes, and this number is expected to rise to 642 million by the year 2040 [2,3]. This increase in DM cases is primarily due to the increase in the number of T2DM incidents that account for as much as 90% of all diabetes cases. T2DM is a highly prevalent and chronic metabolic disorder caused by insufficient insulin production from pancreatic islet β-cells in the setting of insulin resistance, with the inability of cells in muscle, liver, and fat to adequately respond to normal insulin levels. Although T2DM was considered an adult-onset malady for a long time, recent years have witnessed a dramatic increase in the number of juvenile-onset T2DM cases closely associated with the epidemic of childhood obesity [4,5,6,7]. For example, in China, the prevalence of prediabetes and T2DM among children aged 7–17 years increased from 5.2% in 1991 to 13.2% in 2006 [8,9]. In USA, T2DM predominantly affects children from racial and/or ethnic minority groups, such as African American, American Indian, Asian, Hispanic, and Pacific Islander [10,11,12,13,14].

At the molecular level, a subset of transcription factors (TFs) in β-cell—such as musculoaponeurotic fibrosarcoma (MAF) bZIP transcription factor A (MAFA), pancreas/duodenum homeobox protein 1 (PDX1), and homeobox protein NKX6.1—were recently shown to be inactivated under T2DM stress conditions in human T2DM islet β-cells and mouse models of T2DM [15,16,17]. The vital role of various proteins in T2DM pathogenesis is further supported by data reported in the KEGG database resource (available online: http://www.genome.jp/kegg/) [18] which proves information on 24 proteins experimentally shown to be involved in T2DM.

**Figure 1:**
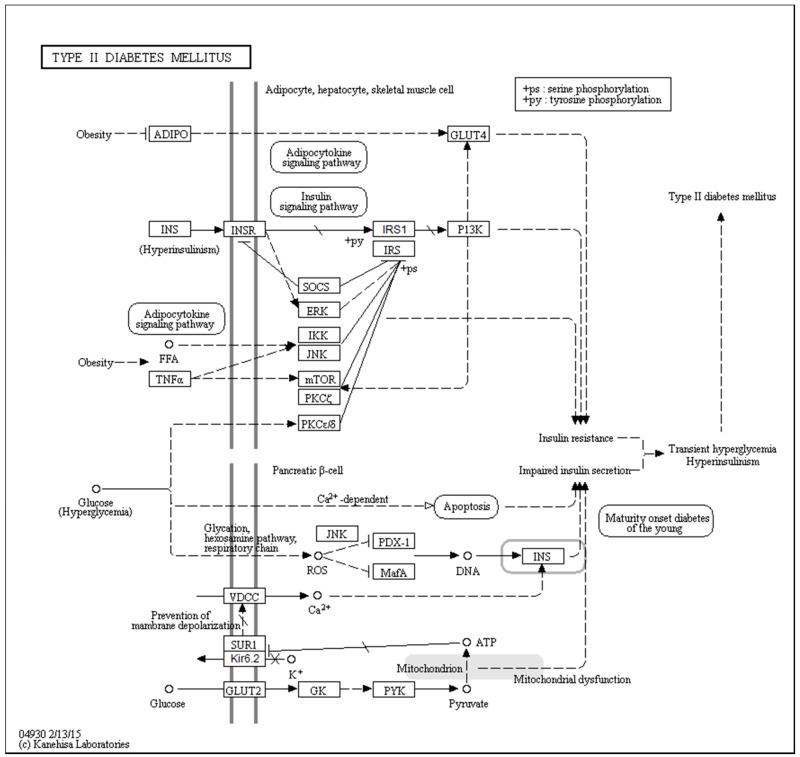
KEGG-based protein–protein interaction network of proteins related to the T2DM pathogenesis (hsa04930). Proteins listed in this network include adiponectin (ADIPO), insulin (INS), insulin receptor (INSR), suppressors of cytokine signaling (SOCS), extracellular signal-regulated kinase 2 (ERK), inhibitor of nuclear factor κ-B kinase (IKK), c-Jun N-terminal kinase (JNK), tumor necrosis factor α (TNFα), mammalian target of rapamycin (mTOR), protein kinase C ξ type (PKCξ), protein kinase C ε/δ type (PKCε/δ), insulin receptor substrate 1 (IRS1), insulin receptor substrate (IRS), phosphatidylinositol 3-kinase (P13K), voltage-dependent L-type calcium channel subunit α-1C (VDCC), sulfonylurea receptor 1 (SUR1), inward rectifier K^+^ channel Kir6.2 (Kir6.2), glucose transporter type 2 (GLUT2), glucokinase (GK), protein-tyrosine kinase (PYK), pancreas/duodenum homeobox protein 1 (PDX-1), and MAF bZIP transcription factor A (MafA). Copyright permission from KEGG Database.

Figure 1 represents a KEGG-based T2DM protein–protein interaction network showing the interplay of proteins involved in several pathways related to the T2DM. Therefore, to better understand their role in the T2DM, a focused look on these proteins is needed. In a recent study, the subcellular localization, function, and likely stability of the β-cell enriched transcription factors were found to be made up of T2DM [17]. The loss of these central factors leads to significant reductions in the expression of their gene targets, including insulin, solute carrier family 2, facilitated glucose transporter member 2 (SLC2A2), and glucagon-like peptide 1 receptor (GLP1R), all of which are critical to β-cell glucose-sensing and insulin secretion [17].

On a more general note, the broad involvement of intrinsically disordered proteins (IDPs) and protein regions (IDPRs) in the pathogenesis of several human diseases, such as amyloidosis, cardiovascular disease, cancer, diabetes, and neurodegeneration, was pointed out, and a D^2^ (disorder in disorders) concept was introduced [19]. It is recognized now that IDPs and IDPRs are very common in nature, and are especially abundant in eukaryotic transcription factors [18,19]. Often, these proteins have critical roles in the regulation of transcriptional machinery [20,21]. The intrinsic lack of structure in IDPs and IDPRs provides several functional advantages that make them ideally suited for mediation of the processes related to transcriptional regulation. These include (i) the presence of small recognition motifs that fold upon binding, (ii) structural flexibility enabling interaction with multiple (often unrelated) targets, (iii) the presence of accessible sites for various post-translational modifications (PTMs) that enables IDPs to function as molecular switches and rheostats, (iv) efficient utilization of a small number of residues to mediate binding interactions, and (v) the ability to bind with high specificity but modest affinity (an important attribute that could facilitate spontaneous dissociation or displacement after signaling is complete) [21,22,23,24,25].

In this paper, we focused on a group of T2DM-related proteins from the KEGG database to gather intrinsic disorder-related information based on the computational analyses of their sequences, and consider these findings in light of the available data on the experimentally validated disordered regions of these proteins and their functions. To this end, we analyzed the intrinsic disorder propensity and the presence of disorder-based functional sites in these proteins by utilizing a series of bioinformatics tools, and also conducted an extensive literature search. The resulting work not only reports and discusses new data and information generated as a result of conducted computational analyses, but also integrates and discusses significant amounts of literature data. Therefore, this study represents a comprehensive survey dedicated to the description of the major biological functions of highly disordered proteins associated with T2DM.

## 2. Results and Discussion

### 2.1. The Overall Intrinsic Disorder Status of the Type 2 Diabetes Mellitus (T2DM)-Related Proteins

**Figure 2:**
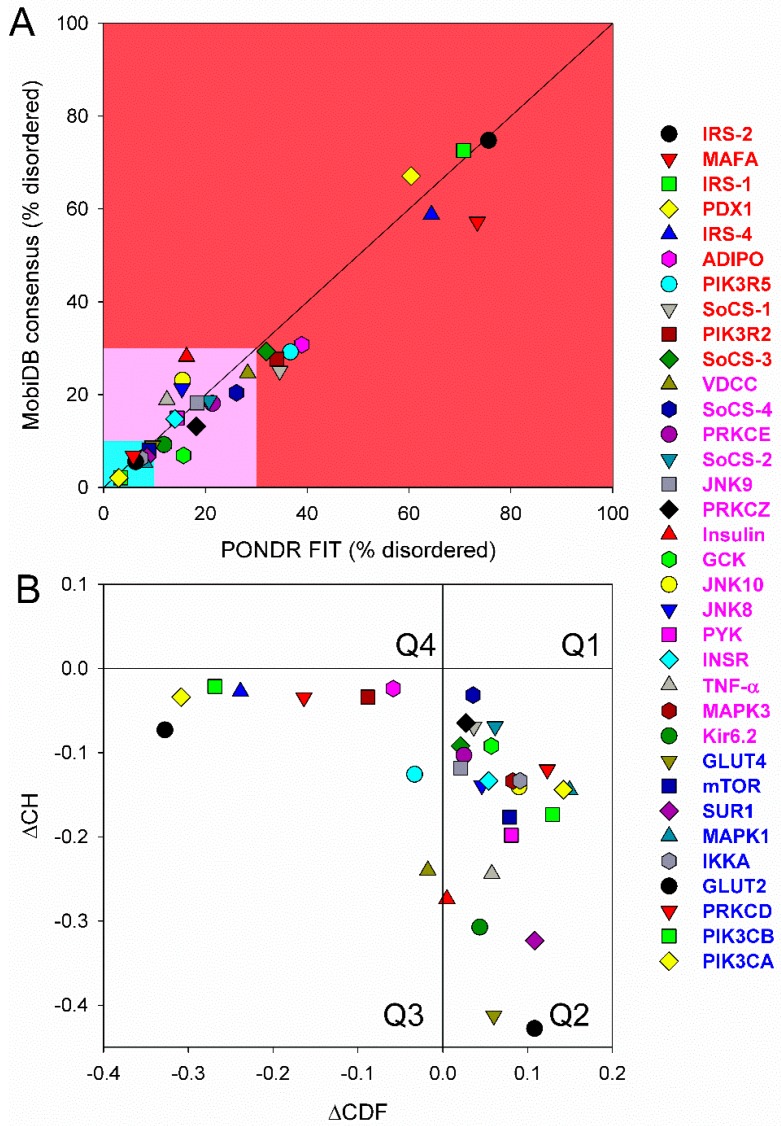
(**A**) Abundance of intrinsic disorder in human T2DM-related proteins. Consensus MobiDB vs. PONDR^®^ FIT plot representing the correlation between the disorder content evaluated by PONDR^®^ FIT (X-axis) and by consensus MobiDB (Y-axis). Solid black line corresponds to the diagonal. Following the accepted practice, two arbitrary cutoffs for the levels of intrinsic disorder are used to classify proteins as highly ordered (PIDRe < 10% light blue field), moderately disordered (10% ≤ PIDR < 30%, light pink field) and highly disordered (PIDR ≥ 30%, light red field) [28]. (**B**) Evaluating intrinsic disorder in human T2DM-related proteins by combined binary disorder classifiers, CH-plot [30], and CDF [30,31,32]. Here, the coordinates of each point were calculated as a distance of the corresponding protein in the CH-plot from the boundary (Y-coordinate) and an average distance of the respective CDF curve from the CDF boundary (X-coordinate). The four quadrants correspond to the following predictions: Q1, proteins predicted to be disordered by CH-plots, but ordered by CDFs; Q2, ordered proteins; Q3, proteins predicted to be disordered by CDFs, but compact by CH-plots (i.e., putative molten globules or hybrid proteins); Q4, proteins predicted to be disordered by both methods.

First, we conducted global analysis of the intrinsic disorder predisposition of 34 T2DM-related proteins from the KEGG database (see Figure 1 and Table S1). To this end, we looked at their overall content of residues predicted to be disordered by two independent per-residue mega-predictors, PONDR^®^ FIT [20] and MobiDB [26,27]. Results of this analysis are summarized in Table S1 (see Supplementary Materials) and shown in Figure 2 as the dependence of the percentage of disordered residues evaluated for different T2DM-related proteins by the MobiDB consensus output on the percentage of disordered residues predicted for these proteins by PONDR^®^ FIT. Figure 2A shows that there is a remarkable agreement between the outputs of these two meta-predictors. This agreement provides a strong support to the validity of the generated evaluations. Furthermore, data presented in Table S1 and Figure 2A clearly indicate that, according to the accepted classification where two arbitrary cutoffs for the levels of intrinsic disorder (percentage of intrinsically disordered residues, PIDR) are used to classify proteins as highly ordered (PIDR < 10%), moderately disordered (10% ≤ PIDR < 30%), and highly disordered (PIDR ≥ 30%) [28], the majority of T2DM-related proteins analyzed in this study are highly or moderately disordered. The aforementioned classification of proteins as highly ordered and moderately or highly disordered is visualized in this plot as light blue, light pink, and light red areas, respectively. Here, IRS2, MAFA, IRS1, PDX1, IRS4, ADIPO, PIK3R5, SoCS1, PIK3R2, and SoCS3 were predicted as highly disordered, VDCC, SoCS4, PRKCE, SoCS2, JNK9, PRKCZ, insulin, GCK, JNK10, JNK8, PYK, INSR, TNF-α, MAPK3, and Kir6.2 were classified as moderately disordered proteins, whereas GLUT4, mTOR, SUR1, MAPK1, IKKA, GLUT2, PRKCD, PIK3CB, and PIK3CA were predicted as mostly ordered.

Note that proteins in this list are ordered according to their content of disordered residues (i.e., residues with the disorder propensity ≥ 0.5) evaluated by the PONDR^®^ FIT (see Table S1). Therefore, 29.4, 44.1, and 26.5% of T2DM-related proteins from the KEGG database are expected to be highly disordered, moderately disordered, and highly ordered, respectively. Furthermore, analysis of the intrinsic disorder predisposition using the PONDR portal (available online: http://pondr.com/) providing an access to the PONDR^®^ VLXT [21], PONDR^®^ VSL2 [22,23,29], and PONDR^®^ VL3 algorithms [24] revealed that 32 of these 34 proteins (94.1%) had intrinsically disordered regions longer than 30 residues, and 13 of the T2DM-related proteins from the KEGG database (38.2%) possessed IDPRs longer than 100 residues, as evaluated by at least one of the three aforementioned PONDR predictors. It is of interest to compare the overall disorder level in the T2DM-associated proteins with the global disorder status of proteins related to other human diseases. In a previous study, it has been established that the majority of the 1,786, 487, 689, and 285 human proteins associated with cancer, cardiovascular disease (CVD), neurodegenerative disease, and diabetes, respectively, are expected to contain long IDPRs [19]. In fact, 79.2 ± 5.2%, 56.9 ± 4.1%, 51.2 ± 4.2%, and 64.5 ± 5.3% of these proteins were predicted to have IDPRs longer than 30 residues, and 26.8 ± 5.6%, 9.2 ± 2.9%, 8.5 ± 2.9%, and 9.6 ± 3.2% of these proteins were shown to have very long IDPRs, with length exceeding 100 residues [19]. It can be seen that the corresponding values retrieved for the T2DM-related proteins from the KEGG database noticeably exceed those obtained in the earlier analysis of proteins associated with cancer, CVD, neurodegenerative disease, and diabetes, clearly indicating that almost all proteins experimentally shown to be related to T2DM contain significant levels of intrinsic disorder. Furthermore, even in the cases when the overall PIDR levels are not too high (e.g., see proteins at the bottom of Table S1), the intrinsically disordered residues are grouped into long IDPRs, indicating their potential functional importance.

Next, we looked into the nature of disorder in human T2DM-related proteins using the binary charge-hydropathy–cumulative distribution function (CH-CDF) classifier. Figure 2B represents the results of this analysis and clearly shows that, according to their localization within the CH-CDF phase space, these proteins can be grouped into two classes: potential native molten globules or hybrid proteins; i.e., proteins predicted to be disordered but compact by CDF and CH analyses, respectively (IRS2, MAFA, IRS1, PDX1, IRS4, ADIPO, PIK3R5, PIK3R2, and VDCC), and proteins expected to be ordered as whole (remaining proteins in the set). There is no single protein in this set which would be predicted to be disordered by both predictors (i.e., none of these proteins is expected to behave as native coil or native pre-molten globule). None of the proteins are predicted as ordered by CDF and disordered by CH-plot analysis. The fact that all T2DM-related proteins analyzed in this study are located below the CH-plot boundary, (i.e., characterized by negative ΔCH scores) suggests that these proteins contain significant levels of hydrophobic residues.

The major goal of the study below is to provide a detailed consideration for several highly disordered proteins known to be associated with T2DM pathogenesis. We discuss some of the most well-studied highly disordered proteins related to T2DM (such as MAFA, IRS, and PDX), and also present intrinsic disorder-related information together with the information on the experimentally validated disordered regions and functions of T2DM proteins from the KEGG database with the highest levels of intrinsic disorder.

### 2.2. Musculoaponeurotic Fibrosarcoma (MAF) bZIP Transcription Factor A (MAFA, UniProt ID: Q8NHW3, PONDR^®^ FIT: 73.2%)

#### 2.2.1. Domain Structure of MAF Proteins

Musculoaponeurotic fibrosarcoma (MAF) proteins are an important class of transcription factors that play essential roles in cell differentiation. The murine MAFA transcription factor is a key regulator of postnatal islet β-cell activity, affecting transcription and secretion of insulin, and regulating β-cell mass [33,34,35]. There are two subgroups of the MAF family: large MAF transcription factors and small MAF transcription factors. The subgroup of large MAFs consists of four proteins: MAFA [36], MAFB [37], c-MAF [38], and Nrl (neural retina-specific leucine) [39], with a wide spectrum of biological activities. For example, MAFA is a β cell-specific transcription activator, which is critical for insulin gene expression [35]. It is reported that MAFA activates the insulin gene C1 element, contributing to the β-cell function and differentiation [35,40,41]. Human *MAFA* expression is known to be markedly decreased in islet β-cells of T2DM patients [42].

**Figure 3:**
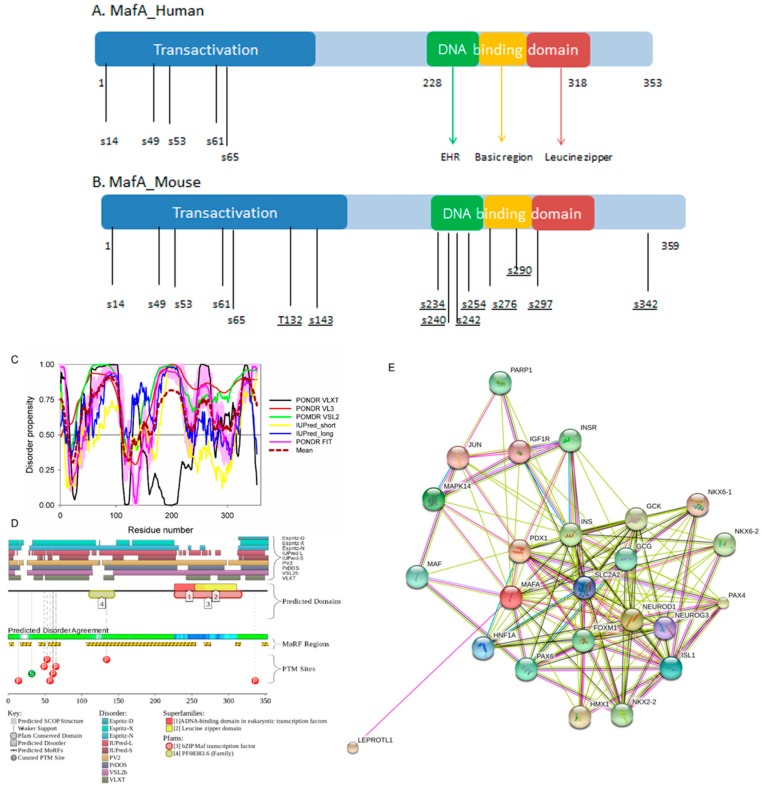
(**A**,**B**) show domain structure of two large MAFs, human and mouse MAFA proteins. In this diagram, the transactivation domain, the extended homology region, the basic region, and the leucine zipper domain are shown by blue, green, yellow, and red, respectively. Positions of known phosphorylation sites are shown. (**C**–**E**) represents the results of the multiparametric analysis of functional intrinsic disorder and interactability of human MAFA protein (UniProt ID: Q8NHW3). (**C**) Evaluating intrinsic disorder propensity by series of per-residue disorder predictors. Disorder profiles generated by PONDR^®^ VLXT, PONDR^®^ VSL2, PONDR^®^ VL3, IUPred_short, IUPred_long, and PONDR^®^ FIT, are shown by black, red, yellow, green, blue, and pink lines, respectively. Dark red dashed line shows the mean disorder propensity calculated by averaging disorder profiles of individual predictors. Light pink shadow around the PONDR^®^ FIT shows error distribution. In these analyses, the predicted intrinsic disorder scores above 0.5 are considered to correspond to the disordered residues/regions, whereas regions with the disorder scores between 0.2 and 0.5 are considered flexible. (**D**) Analysis of the intrinsic disorder propensity and some important disorder-related functional information generated for human MAFA by the D^2^P^2^ database (available online: http://d2p2.pro/) [49]. Here, the green-and-white bar in the middle of the plot shows the predicted disorder agreement between nine predictors, with green parts corresponding to disordered regions by consensus. The yellow bar shows the location of the predicted disorder-based binding sites (molecular recognition features, MoRFs), whereas colored circles at the bottom of the plot show location of various PTMs. (**E**) Analysis of the interactivity of human MAFA by STRING computational platform that produces the network of predicted associations for a particular group of proteins [68].

All the MAF proteins are characterized by a basic region-leucine zipper (bZip) structure. Figure 3A,B represents the domain organization of human and mouse MAFA proteins, which are 353 or 359 residue-long, respectively. Both proteins can be divided into the N-terminal transactivation domain and the C-terminal DNA binding bZip domain. In its turn, the highly conserved DNA-binding bZIP domain consists of a basic region and a leucine zipper motif. The basic region was named for the series of positively charged residues that interacts with DNA, whereas the leucine zipper (or leucine scissors) dimerization region, which is a subtype of coiled coil domains, is characterized by the presence of leucine residues at every seventh position, which pack against each other every second turn of the α-helices. MAF proteins show high conservation in the extended homology region (EHR) or ancillary DNA binding region, a small domain N-terminal to a basic amino acid rich region. The EHR in combination with the basic region defines the DNA binding specificity of MAF proteins. In fact, the DNA sequence recognized by the MAF proteins includes 13–14 base pairs, which is longer than the 8 base pair core binding site of other bZIP factors, such as AP1 and CREB [43].

#### 2.2.2. Intrinsic Disorder Status of Human MAFA Protein

Previous computational studies have found that IDPRs are generally more prevalent in transcription factors than would be expected by chance, especially in eukaryotes [44,45,46]. It is likely that intrinsic disorder in MAFA has several functional implications relating this protein to the T2DM pathogenesis. For example, it might help this protein to act as a transcriptional activator, promoting specific binding of MAFA to the insulin enhancer element RIPE3b, and thereby activating insulin gene expression. MAFA cooperates synergistically with NEUROD1 and PDX1. Phosphorylation by GSK3 increases its transcriptional activity and is required for its oncogenic activity. Furthermore, in addition to its important roles in T2DM, MAFA can serve as either an oncogene or a tumor suppressor, depending on cell context [47].

To illustrate the prevalence and functional roles of intrinsic disorder in human MAFA protein, Figure 3C represents the outputs of several computational tools for predicting the per-residue disorder propensities of a query protein (PONDR^®^ FIT, PONDR^®^ VLXT, PONDR^®^ VL3, and PONDR^®^ VSL2 algorithms [20,21,23,29,48], IUPred_long and IUPred_short [25]).

This multiparametric computational analysis conducted in our study revealed that human MAFA is predicted to possess a high level of intrinsic disorder. In fact, Figure 3C shows that almost the entire protein is predicted to be disordered by the majority of computational tools used in this study. The only exception is the PONDR^®^ VLXT output, showing that the central 130 or so residues of this protein (residues 110–240) have some reasonable tendency for order. Curiously, the majority of this region (residues 147–220) is characterized by computational bias and is described as a His-rich region.

Figure 3D represents an image generated by the D^2^P^2^ database (available online: http://d2p2.pro/) [49], which, in addition to the outputs of several disorder predictors (IUPred [25], PONDR^®^ VLXT [20], PrDOS [50], PONDR^®^ VSL2B [24,48], PV2 [49], and ESpritz [51]) contains information on the potential functionality of IDPRs in a query protein. In fact, Figure 3D indicates that MAFA contains multiple sites of various posttranslational modifications (PTMs), which is in agreement with the well-known fact that phosphorylation [22] and many other enzymatically catalyzed PTMs are preferentially located within the IDPRs [52]. It is known that many IDPRs contain local regions with a strong tendency to become ordered upon interaction with specific binding partners; i.e., specific recognition regions that can undergo coupled folding and binding [53,54,55,56,57] and therefore might serve as the disorder-based potential binding sites [58]. There are several predictors for finding such molecular recognition features (MoRFs) [59,60,61,62] and the results of application of one of them, ANCHOR [61,62], are shown in Figure 3D.

It is generally accepted to represent protein–protein interactions (PPIs) in a form of a network, where each protein is presented by a node and interaction is shown as a link between the corresponding nodes. One of the parameters describing the network topology is the degree (or connectivity) of a node, which reflects the number of nodes to which a query node is connected. Nodes with high degree (e.g., nodes that are in the top 20% of the degree distribution [63]) are called hubs. There are several criteria used for identifying hubs in PPI networks, with one of the robust hub definitions being a protein with at least five interactions [64,65,66,67]. In line with this PPI network concept, Figure 3E represents an interactome of MAFA generated by the STRING (Search Tool for the Retrieval of Interacting Genes) platform (available online: http://string-db.org/) [68], which shows the predicted and experimentally-validated interaction partners of this protein, and indicates that MAFA serves as an important and highly disordered hub protein. In agreement with this observation, Table S1 shows that according to the Agile Protein Interactomes DataServer (APID) platform [69], MAFA is engaged in at least 12 interactions with other human proteins. As a matter of fact, Table S1 illustrates that high interactability represents a common feature of all the T2DM-related proteins analyzed in this study. The number of APID-identified interactions per a T2DM-related protein ranges from 6 to 386, and altogether these proteins interact with 3439 partners, generating a value of 101.15 interactions per protein.

Since the complete human interactome remains unknown, this average value (as well as values for all human proteins associated with T2DM listed in Table S1) can be compared with the average numbers of experimentally established interactions reported for human proteins assembled in several PPI network. For example, in a PPI network consisting of 3186 interactions among 1705 human proteins that was created by screening of a protein matrix of 4456 baits and 5632 preys by automated yeast two-hybrid interaction mating, proteins were reported to have on average 1.87 interaction partners, with 804 proteins interacting with just one partner, and with 24 hub–proteins interacting with more than 30 partners each [70]. According to the APID server [69], 11,380 human proteins are engaged in 63,667 interactions proven by at least two independent publications, indicating that on average, one human protein might have 5.59 interaction partners. Finally, a recently released BioPlex 2.0 (biophysical Interactions of ORFeome-derived complexes) platform, which was assembled utilizing information derived from the affinity purification–mass spectrometry data [71], reports 56,553 interactions between 10,961 human proteins; i.e., describes a PPI network with 5.16 interactions per protein. Obviously, the average number of interactions per T2DM protein (101.15) notably exceeds those evaluated for various human PPIs (ranging from 1.87 to 5.59), clearly indicating that the vast majority of diabetes-related proteins analyzed in this study are highly connected and therefore can be considered as important hubs.

Overall, Figure 3 provides a clear picture of both high prevalence and functional importance of intrinsic disorder in human MAFA protein. The literature data considered below provide support to the notion of the importance of intrinsic disorder for MAFA functionality.

#### 2.2.3. Functionality of Intrinsic Disorder in Human MAFA Protein

**Figure 4:**
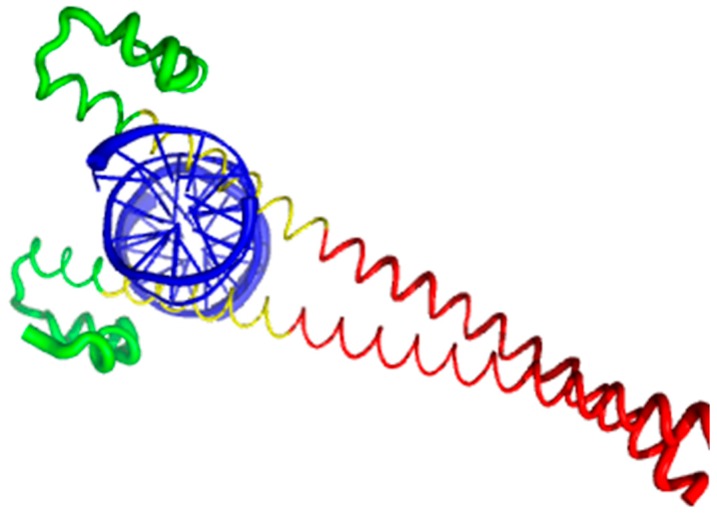
Crystal structure of the complex between the musculoaponeurotic fibrosarcoma (MAF) bZIP transcription factor A (MAFA) and MAF recognition element (MARE) (PDB entry 4EOT), where the flexibility of different protein regions is shown by varying the thickness of the backbone (based on the B-factor values). Here, more flexible regions are shown by thicker backbone. As can be expected, the loop areas that point toward the aqueous environment are the most flexible regions of this domain in its bound form. The extended homology region (EHR), the basic region, and the leucine zipper domain are shown by green, yellow, and red color, respectively. DNA molecule is shown by blue color. The EHR of MAFA is mobile, as reflected by high B factors.

Currently, structural information is only available for the portion of the C-terminal DNA-binding domain of human MAFA (residues 226–318). Crystal structure was determined for this MAFA region in a complex with a 19-mer duplex DNA containing the 13 bp MAF recognition element (T-MARE) consensus sequence and Hoogsteen end-to-end packing of the DNA (PDB ID: 4EOT) [72]. Figure 4 shows that in the DNA bound form, this domain exists as a well-folded dimer containing an extended homology region (EHR), a basic region, and a leucine zipper domain. To illustrate the differences in the conformational mobility of different parts of this domain, Figure 4 uses different thicknesses of the backbone to show the distribution of B-factor within the DNA-binding domain. 

It can be seen that the basic helix of MAFA is ordered. Arg 260 is embedded into the most stable region of MAFA. The MAFA-specific EHR, which extends the basic helix and forms two additional short helices, appears to stabilize the basic helix via hydrogen bonding and van de Waals interactions with the basic region [73]. The interactions between the phosphoamino acid-rich transactivation and b-Zip domain possibly control MAFA DNA-binding activity and create distinctive interfaces for coregulatory proteins [74]. The EHR does not contact DNA directly but stabilizes DNA binding by contacting the basic helix. It was also suggested that the DNA binding process involves a conformational change from contacting the core-TGA to contacting the flanking-TGC bases [73].

Although the structure of the MAFA DNA-binding domain presented in Figure 4 seems to contradict the results of the computational evaluation of the intrinsic disorder propensity of human transcription factor MAFA (Figure 3 shows that most of the protein, including its DNA-binding domain, are expected to be disordered), it is worth keeping in mind that this structure was solved for the MAFA-DNA complex. In fact, earlier analysis revealed that the unbound form of this DNA-binding domain (residues 227–301 of the human MAFA) is intrinsically disordered in solution and undergoes a disorder-to-order transition after interaction with insulin MARE [75]. Furthermore, the basic region (approximately 20 amino acids) is intrinsically disordered [76,77], and its folding is coupled with DNA binding, which also induces a conformational change in the DNA molecule [78,79]. Similarly, the dimerization leucine zipper region forms two α-helices that are wound around each other to form a supercoil in the homo-dimeric form, but is mostly disordered in the un-bound form. Therefore, foldable intrinsic disorder regions are crucial for the DNA binding function and dimerization of the transcription factor MAFA.

Furthermore, MAFA proteins appear to be heavily phosphorylated [41,80], which impacts their roles in transactivation [81,82] and DNA binding [83]. In fact, phosphorylation is one of the important regulatory mechanisms controlling the structure and function of MAFA. This PTM impacts protein stability [41,72,81], transactivation activity [72,81,82], and DNA binding [84]. The N-terminal region of the large MAFA proteins is a transactivation domain rich in phosphorylation sites. This domain has two serine residues (S14 and S65) which are important for its transcriptional capacity, and control the expression of differentiation programs in NR cells [82,85]. The phosphorylation at serine (S65) in MAFA is necessary for ubiquitin-mediated degradation [41]. In addition, S65 phosphorylation in MAFA (and likely MAFB) is required for priming Glycogen Synthase Kinase 3 (GSK3) for phosphorylation at S61, threonines T57 and T53, and S49, which enhances the transactivation and transformation potential [41]. Figure 3 shows that all these PTM sites are located within the IDPRs of human MAFA protein, indicating the important role of intrinsic disorder in PTM-driven regulation of the functionality of this protein.

### 2.3. Insulin Receptor Substrates, IRS1 (UniProt: P35568, PONDR^®^ FIT: 70.7%), IRS2 (UniProt: Q9Y4H2, PONDR^®^ FIT: 75.6%), and IRS4 (UniProt: O14654, PONDR^®^ FIT: 64.4%)

Insulin receptor substrates (IRSs) are a family of cytoplasmic adaptor proteins that link signaling from upstream activators to multiple downstream effectors to modulate normal growth, metabolism, survival, and differentiation of the cell. They mediate many of the key metabolic actions of insulin. The IRS family has four known members: IRS1, IRS2, IRS3, and IRS4. IRS1 and IRS2 are widely expressed in most human tissues and are the primary mediators of insulin dependent mitogenesis and regulation of glucose metabolism [86]. A third protein, IRS4, is found primarily in brain, kidney, thymus, and liver [87], whereas IRS3 has so far only been identified in rodents. IRS1, IRS2, and IRS4 with their CDRFIT (content of disordered residues predicted PONDR^®^ FIT) values of 70.7, 75.6, and 64.4% respectively, are among the most disordered proteins in the KEGG dataset of proteins related to T2DM.

IRSs are cytoplasmic scaffolding proteins that mediate signaling between multiple receptor tyrosine kinases (RTK), including the insulin-like growth factor I (IGF1) and insulin receptors, and several other Src homology 2 (SH2) domain-containing proteins [88,89]. It is identified that at least 50 proteins associate with IRSs, including proteins modulating tyrosine phosphorylation of IRSs by receptor kinase, proteins controlling stability of IRSs, proteins determining intracellular localization of IRSs, and proteins mediating insulin-like activities [90]. In addition, it has been identified that IRS1 interacts with RNA molecules [91,92]. The intrinsic disorder of IRSs may allow them to fold at binding and structurally accommodate multiple interaction partners.

#### 2.3.1. Domain Structure of the Insulin Receptor Substrates

**Figure 5:**
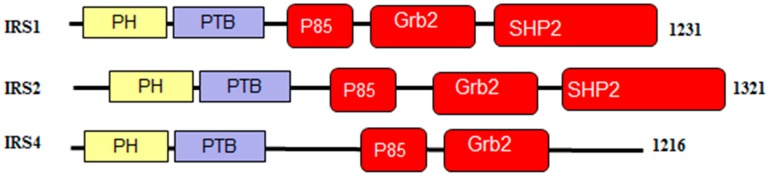
Schematic representation of the signaling domains in the proteins from human IRS family. Here, PH, is Pleckstrin homology domain; PTB, is the phosphotyrosine-binding domain; Src homology region 2-containing protein tyrosine phosphatase 2 (SHP2) binding domain is present in IRS1 and IRS2, and is not found in IRS4.

As shown in Figure 5, IRS1, IRS2, and IRS4 proteins consist of an amino terminal pleckstrin homologydomain (PH) and a phosphotyrosine-binding domain (PTB) and N-terminal tyrosine residues that act as phosphorylation sites to drive insulin/IGF-1 signaling [93]. The p85 and GRB2 domains contain phosphorylatable tyrosine residues which are used to bind SH2-containing effector proteins for the activation of the PI3K/AKT and MAPK/ERK signaling pathways [89,93,94]. Furthermore, the C-terminal regions of IRS1 and IRS2 have an additional binding site for interaction with the SH2 domain-containing protein, tyrosine phosphatase-2 (SHP2), providing a negative feedback loop by dephosphorylating the tyrosine residues responsible for binding [95,96,97,98,99,100].

#### 2.3.2. Order and Intrinsic Disorder in Functionality of Human IRS1, IRS2, and IRS4 Proteins

IRS1 (UniProt ID: P35568) contains multiple tyrosine phosphorylation sites, which, during insulin stimulation, are phosphorylated and act as docking sites for multiple SH2-containing proteins, such as PI3K, Grb2, Nck, Crk, Fyn, Syp, and SHP2 [93]. For instance, the phosphorylation of Tyr612 and Tyr632 generates docking sites for the phosphatidylinositol 3-kinase (PI3K) [101]. Interaction with these sites activates the downstream PI3K pathway and leads to the glucose uptake and glycogen synthesis [102,103]. S6K directly phosphorylates IRS1 on Ser270, Ser307, Ser636, and Ser1101 to promote insulin resistance in response to the tumor necrosis factor (TNF)-α signaling through inhibitor of nuclear factor kappa-B kinase subunit β (IKK2) [104,105].

**Figure 6:**
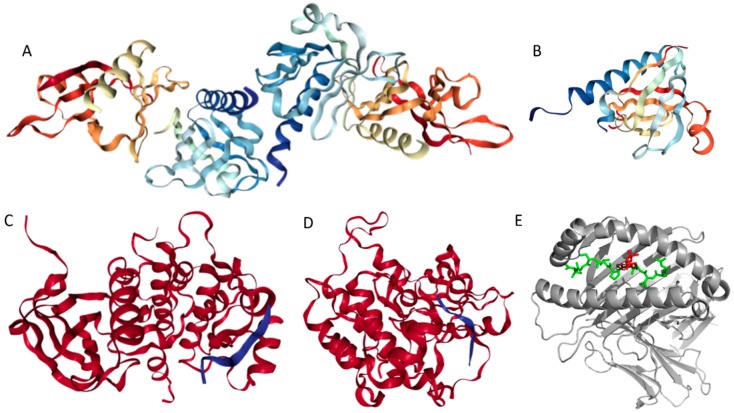
Structural information available for the members of human IRS family. (**A**) Crystal structure of the dimeric form of the PH-PTB targeting region of human IRS1 (residues 4–267, PDB ID: 1QQG). (**B**) NMR structure of the PTB domain of IRS1 (residues 157–267) complexed with a tyrosine-phosphorylated peptide derived from the IL-4 receptor (PDB ID: 1IRS). (**C**) Crystal structure of the GRB2-binding region of human IRS1 (residues 891–902, blue ribbon) complexed with the protein kinase domain of the insulin-like growth factor 1 receptor (red cartoon) (PDB ID: 1K3A). (**D**) Crystal structure of a short peptide of human IRS1 (residues 731–736, blue ribbon) complexed with the insulin receptor (red cartoon) (PDB ID: 2Z8C). (**E**) The IRS2 peptide (residues 1097–1105, RVASPTSGV, green structure) complexed with the A-2 α chain of human leukocyte antigen (HLA) class I histocompatibility antigen, HLA A2 (PDB ID: 3FQW).

**Figure 7:**
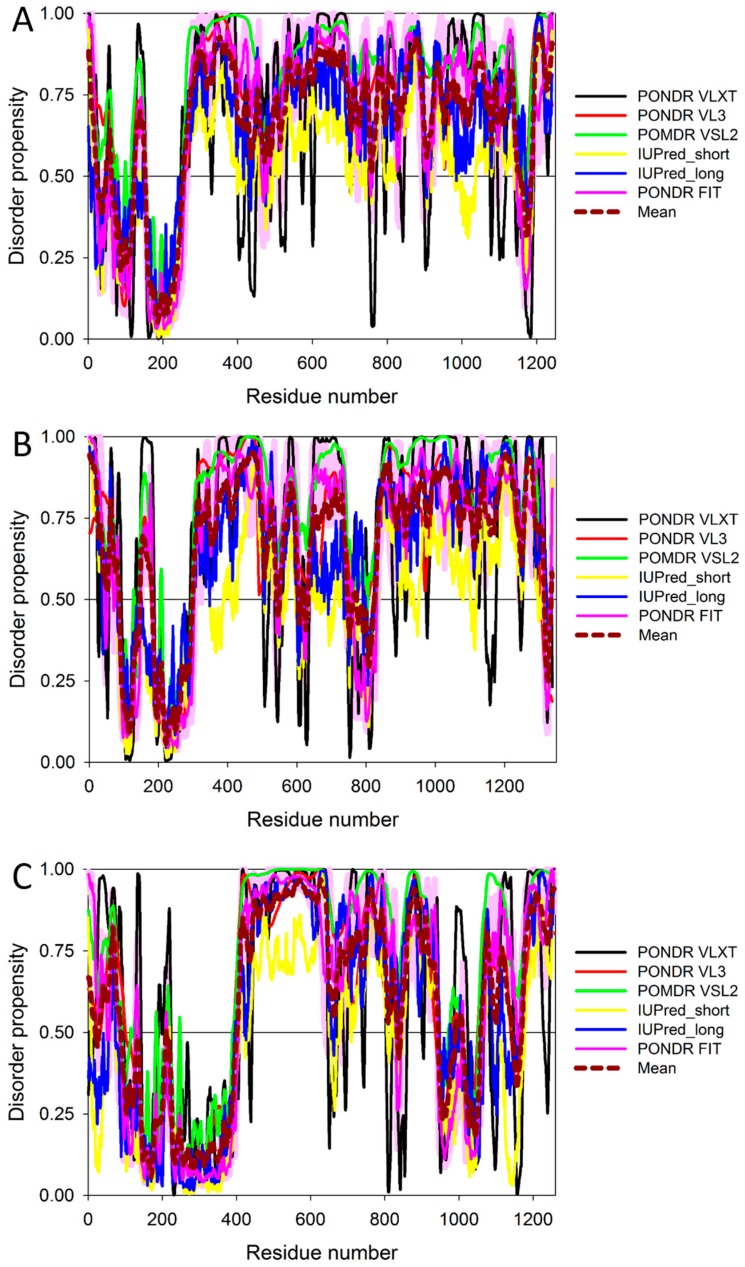
Multiparametric analysis of the abundance of intrinsic disorder in human IRS1 (UniProt ID: P35568) (**A**), IRS2 (UniProt ID: Q9Y4H2) (**B**), and IRS4 (UniProt ID: O14654) (**C**) by PONDR^®^ VLXT, PONDR^®^ VSL2, PONDR^®^ VL3, IUPred_short, IUPred_long, and PONDR^®^ FIT. Keys are the same as in legend to Figure 5A.

Structures of several regions of human IRS1 were solved (see Figure 6A–D). These include: the dimeric form of the PH-PTB targeting region (residues 4–267, PDB ID: 1QQG, Figure 6A); the PTB domain (residues 157–267) complexed with a tyrosine-phosphorylated peptide derived from the IL-4 receptor (PDB ID: 1IRS, Figure 6B); a short peptide (residues 891–902) corresponding to the GRB2-binding region complexed with the insulin-like growth factor 1 receptor (PDB ID: 1K3A, Figure 6C); and another short peptide (residues 731–736) complexed with the insulin receptor (PDB ID: 2Z8C, Figure 6D). Importantly, the GRB2-binding region of this protein (residues 896–898) is embedded in one of the predicted disorder-based binding sites, MoRFs (residues 894–902, see Table S1). Furthermore, the 731–736 region engaged in interaction with insulin receptor is a part of another MoRF (residues 722–736, see Table S1). In the structure of the PH-PTB dimer (PDB ID: 1QQG), no coordinates have been determined for residues 4–11, 115–158, and 263–267, indicating that they are located within regions of missing electron density and therefore are likely to be characterized by high conformational flexibility. These regions are predicted to be disordered (see Figure 7A below).

Human IRS2 (UniProt ID: Q9Y4H2) is the most disordered protein analyzed in this study (see Table S1 and Figure 2). The crystal structure is known for the IRS2 peptide (residues 1097–1105, RVASPTSGV) complexed with the A-2 α chain of human leukocyte antigen (HLA) class I histocompatibility antigen, HLA A2 (PDB ID: 3FQW).

**Figure 8:**
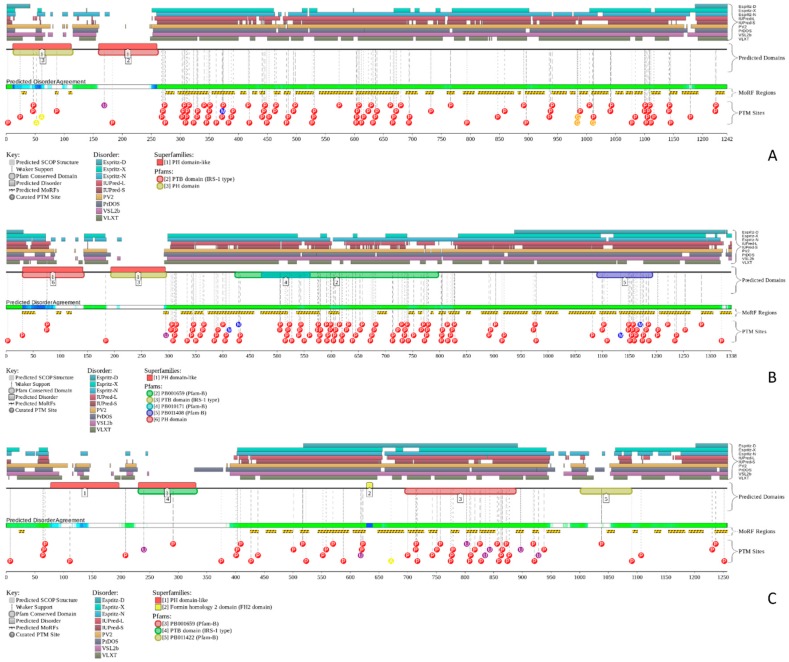
Analysis of the intrinsic disorder propensity and some important disorder-related functional information generated for human IRS1 (**A**), IRS2 (**B**), and IRS4 (**C**), by the D^2^P^2^ database (available online: http://d2p2.pro/). Keys are the same as in legend to Figure 4B.

The phosphorylated IRS2 peptide at the P4 position is accommodated within the HLA A2 binding cleft with little change when compared with the non-phosphorylated counterpart (Figure 6E) [106]. Phosphorylation of P4-Ser in IRS4 leads to an altered peptide conformation attributable to steric constraints. Figure 7B shows that both these regions are predicted to be highly disordered, whereas Figure 8B and Table S1 indicate that they contain multiple predicted disorder-based binding sites (MoRFs).

Human IRS4 (UniProt ID: O14654) is characterized by the lowest disorder content among the members of the human IRS family, and no structural information for this protein is currently available. IRS4 differs from the other IRS proteins by the absence of the SHP2-binding motif. As a resu). Crystal structure of a singleative feedback loop through tyrosine dephosphorylation of IRS1 and IRS2 by SHP2 may not exist for IRS4 signaling [107]. Finally, as shown in Table S1, the MoRFCHiBi web predicts that the regions responsible for the binding CRK (residues 678–800) and for interaction with GRB2 (residues 895–897) are included within MoRF regions spanning from residues 698–706, 741–747, 776–785, 808–811, and 890–900 respectively. This indicates that these IDPRs may help in mediating biological activities of human IRS4 protein.

Figure 7 and Figure 8 show that all three members of the human IRS family are predicted to be highly disordered, but possess N-terminal ordered regions that include the pleckstrin (PH) homology domain (residues 12–115, 16–144, and 78–199 in IRS1, IRS2, and IRS4, respectively) and the IRS-type phosphotyrosine-binding domain (PTB, residues 160–264, 194–298, and 231–235 in IRS1, IRS2, and IRS4, respectively).

Figure 8 indicates that these three proteins are heavily decorated by various PTMs, such as phosphorylation, ubiquitination, acetylation, glycosylation, and methylation (see red, purple, yellow, orange, and blue circles in the bottom parts of the corresponding plots). All these PTMs are preferentially located within the highly disordered central and C-terminal regions of IRS proteins. Furthermore, Figure 8 and Table S1 show that these proteins contain numerous disorder-based binding sites, several of which were experimentally proven to be responsible for interactions with specific binding partners. For example, IDPRs of all three proteins contain multiple YXXM motifs (e.g., residues 465–468, 551–554, 612–615, 632–635, 662–665, 732–735, 941–944, 989–992, and 1012–1015 in IRS1; residues 540–543, 598–601, 653–656, 675–678, 742–745, 823–826, and 1072–1075 in IRS2; residues 487–490, 700–703, 717–720, 743–746, 779–782, 828–831, and 921–924 in IRS4) that are typically utilized for interaction with the SH2 domains of the p85 regulatory subunit of phosphoinositide 3-kinase. The vast majority of these YXXM motifs in all three proteins are located within their numerous MoRFs (see Table S1).

Curiously, it was also indicated that the high-affinity activation of the phosphoinositide 3-kinase requires the simultaneous binding of two phosphorylated YXXM motifs of the IRS1 to the two SH2 domains of the kinase [108]. Furthermore, the 896–898 region of IRS1 and the 895–897 region in IRS4 are needed for the binding of these proteins to the growth factor receptor-bound protein 2 (GRB2), which is an adapter protein providing an important connection between cell surface growth factor receptors and the RAS signaling pathway. Finally, in IRS4, the 678–800 region, which is predicted to contain at least three MoRFs (see Figure 8C and Table S1), is known to be engaged in interaction with the proto-oncogene c-CRK protein (or adapter molecule CRK).

**Figure 9:**
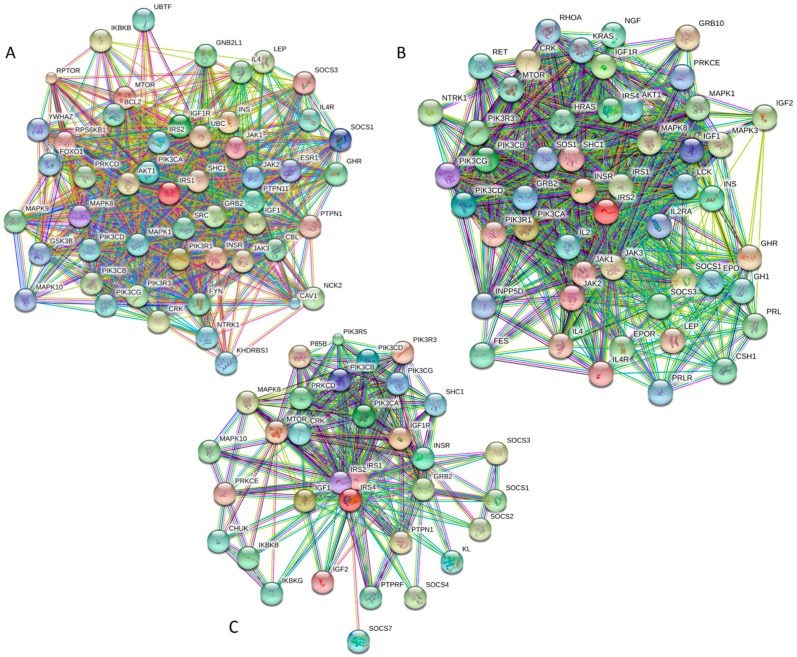
Analysis of the interactivity of human IRS1 (**A**), IRS2 (**B**), and IRS4 (**C**), by STRING computational platform.

Figure 9 and Table S1 provide further support to the idea that human IRS proteins serve as important hubs, being placed at the centers of highly developed protein–protein interaction networks. Table S1 shows that according to APID, human IRS1, IRS2, and IRS4 are engaged in interaction with 107, 58, and 138 partners, respectively. It is likely that this high interactivity of IRS proteins is determined by their highly disordered nature and by the presence of numerous disorder-based binding sites. 

According to the KEGG database, IRS1, IRS2, and IRS4 orthologies (orthology K16172) are engaged in numerous physiological and pathological pathways. Among common pathological pathways are insulin signaling, T2DM, insulin resistance, adipocytokine signaling, aldosterone-regulated sodium reabsorption, regulation of lipolysis in adipocytes, neurotrophin signaling, non-alcoholic fatty liver disease, and MicroRNAs in cancer. Therefore, these proteins are related to the pathogenesis of cancer and several endocrine and metabolic diseases. It is likely that the high intrinsic disorder in these proteins allows them to be engaged in a multitude of signaling and pathological pathways.

In summary, Figure 7, Figure 8, and Figure 9 present the results of the multifactorial computational analysis of the prevalence of functional intrinsic disorder in IRS1, IRS2, and IRS4 proteins, and clearly show that all these proteins are highly disordered and contain numerous PTMs and disorder-based binding sites. It is also evident that these proteins are engaged in numerous protein–protein interactions, often utilizing numerous disorder-based binding sites (see also Table S1).

### 2.4. Pancreatic and Duodenal Homeobox 1 Protein, PDX1 (UniProt ID: P52945, PONDR^®^ FIT: 60.4%)

#### 2.4.1. Domain Structure of the PDX1 Protein

PDX1 (pancreatic and duodenal homeobox 1) is known as a transcription factor that regulates pancreas development and β-cell differentiation. It is shown that both genetic and acquired reductions in *PDX1* expression cause type 2 diabetes, β-cell dysfunction [109,110,111,112,113], and impaired islet compensation in the presence of insulin resistance [109,110]. The content of disordered residues predicted by PONDR^®^ FIT (CDRFIT) is 60.4%. Since the PDX1 is another transcription factor, and since transcription factors are known to possess extended regions of intrinsic disorder [46], it is not too surprising to find that it is predicted to be highly disordered (see Table S1).

**Figure 10:**
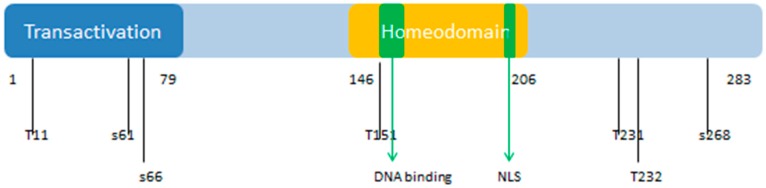
Functional domains and phosphorylation sites of human Pancreatic and Duodenal Homeobox 1 Protein (PDX1). The numbers indicate the positions of phosphorylation sites and functional domains on the polypeptide chain of human PDX1. PTD: Protein transduction domain; S: Serine; T: Threonine. NLS: nuclear localization signal. PDX1 is posttranslationally modified to modulate its function, stability, and subcellular location. Numbers indicate serine (S) and threonine (T) phosphorylation sites and functional domains within the protein. These various sites are phosphorylated by different kinases, which include Thr11 by DNA-PK, Ser61 and Ser66 by GSK3β, Thr152 by PASK, Thr231 and Ser232 by CK2, and Ser268 by AKT-GSK and HIPK2.

The *PDX1* gene codes for a protein with 283 amino acids (see Figure 10). The PDX1 protein sequence is very homologous among different species. There is a transactivation domain at the N-terminus. The middle region contains a homeodomain (residues 146–206), which is responsible for DNA binding and protein–protein interactions in a transcriptional activation mechanism.

The homeodomain region contains a nuclear localization signal (NLS) sequence at residues 197–203 (RRMKWKK), which appears to be important for its DNA binding activity [114]. There is a conserved motif (residues 210 to 238) in the C-terminus of PDX1 that mediates the PDX1-PCIF1 interaction resulting in the inhibition of PDX1 transcriptional activity [115]. Figure 10 also shows the localization of phosphorylation sites within the protein. These various sites are phosphorylated by different kinases, which include Thr 11 by DNA-PK, Ser 61 and 66 by GSK3β, Thr 152 by PASK, Thr 231 and Ser 232 by CK2, and Ser 268 by AKT-GSK and HIPK2 [116]. According to the KEGG database, PDX1 plays a role in insulin secretion, type 2 diabetes mellitus, and maturity onset diabetes of the young pathways. It is also related to the endocrine and metabolic diseases, such as maturity onset diabetes of the young (MODY), permanent neonatal diabetes mellitus (PNDM), and pancreatic agenesis (see K07594 orthology in KEGG database, available online: http://www.genome.jp/kegg/).

#### 2.4.2. Prevalence of Functional Intrinsic Disorder in Human PDX1 Protein

**Figure 11:**
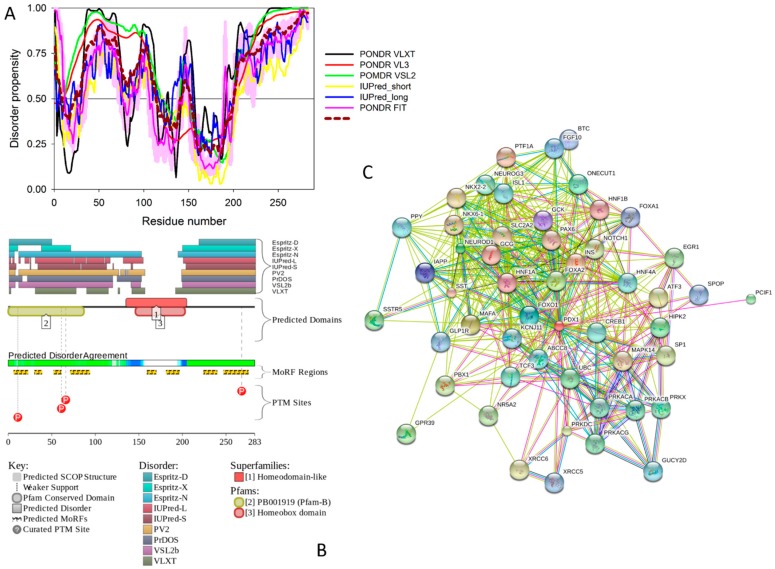
Multiparametric analysis of functional intrinsic disorder and interactability of human PDX1 protein (UniProt ID: P52945). (**A**) Evaluating intrinsic disorder propensity by series of per-residue disorder predictors. Disorder profiles generated by PONDR^®^ VLXT, PONDR^®^ VSL2, PONDR^®^ VL3, IUPred_short, IUPred_long and PONDR^®^ FIT. (**B**) Analysis of the intrinsic disorder propensity and some important disorder-related functional information generated for human PDX1 protein by the D^2^P^2^ database (available online: http://d2p2.pro/). (**C**) Analysis of the interactivity of human PDX1 protein by STRING computational platform.

Figure 11 represents the results of the multifactorial computational analysis of the prevalence of functional intrinsic disorder in human PDX1, and shows that this protein is predicted to be highly disordered and contain numerous PTMs and disorder-based binding sites. Figure 11 shows that homeodomain is predicted to house both ordered and disordered subregions, while the N- and C-terminal regions of PDX1 are predicted to be intrinsically disordered by multiple algorithms. It is expected that regions flanking the homeodomain exhibit significant disorder, which suggests a functional role for such IDPRs in interaction of this TF with DNA [82,83], permitting PDX1 to bind DNA with high affinity but relatively low specificity [117,118]. In addition, the NLS domain and PDX-1-PCIF1 domain all have MoRFs segments (188–201 and 233–265) as shown in Table S1, which may be involved in regulatory interactions with other proteins, ligands, or DNA [119,120]. In fact, Figure 11C shows that human PDX-1 protein is located in the middle of the well-developed PPI. Furthermore, Table S1 indicates that according to APID, this protein can interact with 20 partners, indicating that human PDX-1 represents another example of a T2DM-related intrinsically disordered hub.

### 2.5. Adiponectin (UniProt ID: Q15848; PONDR^®^ FIT: 38.9%)

Adiponectin is a circulating adipokine or an adipose tissue-derived hormone that has important anti-inflammatory and insulin sensitizing properties, and is downregulated in obese individuals [121], as well as in the insulin-resistant humans and animals [122]. Adiponectin modulates several metabolic processes and plays a protective role in the development of insulin resistance, atherosclerosis, and inflammation [123]. The importance of adiponectin for the pathogenesis of T2DM and cardiovascular disease (CVD) is associated with the ability of this adipokine to improve systemic glucose tolerance and to protect the vasculature from atherosclerosis [124]. Therefore, hypoadiponectinemia is a risk factor for the T2DM and CVD development [125]. Furthermore, this abundant adipokine is involved in fatty acid catabolism and glucose regulation [126,127].

In summary, adiponectin is clearly a multifunctional protein with important regulatory roles in anti-inflammatory response (via decreasing the neutrophil adhesion and macrophage activation), cardioprotection (via anti-ischemic function), metabolism (via controlling glucose utilization, insulin sensitivity, and fatty acid oxidation), and vascular protection (via enhancing production of nitric oxide and stimulation of the angiogenesis) [127,128]. The biological activities of this adipokine are controlled by various post-translational modifications, PTMs (e.g., hydroxylation and glycosylation).

**Figure 12:**
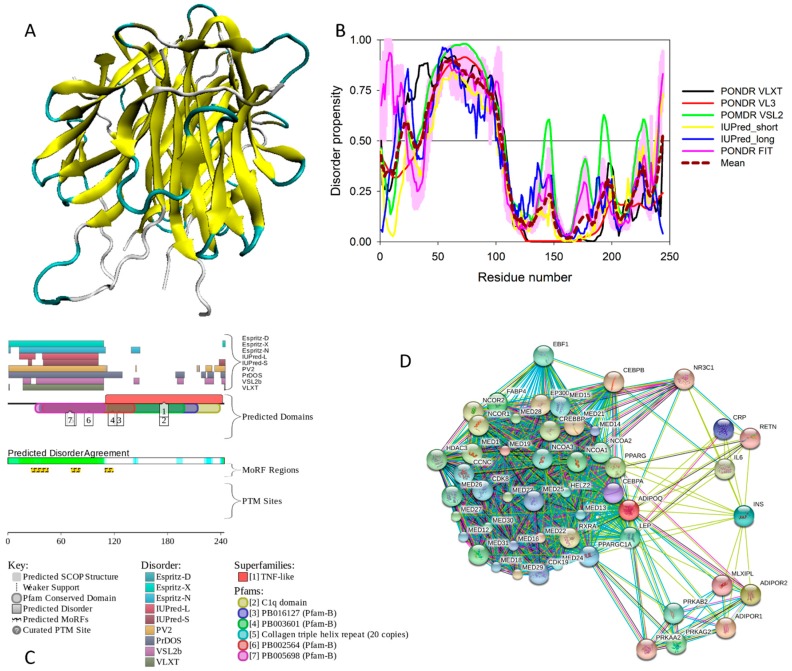
Structure and intrinsic disorder in human adiponectin (UniProt ID: Q15848). (**A**) Crystal structure of a single-chain trimer of human adiponectin globular domain (PDB ID: 4DOU). (**B**) Evaluating intrinsic disorder propensity of human adiponectin by series of per-residue disorder predictors. Disorder profiles generated by PONDR^®^ VLXT, PONDR^®^ VSL2, PONDR^®^ VL3, IUPred_short, IUPred_long, and PONDR^®^ FIT. (**C**) Analysis of the intrinsic disorder propensity and some important disorder-related functional information generated for human adiponectin by the D^2^P^2^ database (available online: http://d2p2.pro/). (**D**) Analysis of the interactivity of human adiponectin by STRING computational platform.

Human adiponectin (UniProt ID: Q15848) is a 244 residue-long protein that is synthesized as a precursor containing signal peptide (residues 1–18) needed for targeting this adipokine for extracellular section and cleaved at maturation. Mature protein has a non-conserved N-terminal domain (residues 19–41), a collagen-like domain (residues 42–107), and a C-terminal globular domain (residues 108–244). The crystal structure of a globular domain of human adiponectin is known (PDB ID: 4DOU, see Figure 12A).

This structure was obtained for a single-chain trimer, where three globular domains encompassing residues Pro104 to Asn244 of human adiponectin were directly joined using the C- and N-terminal residues of the two adjacent monomers and utilizing the flexible Pro104-Tyr109 region as a natural linker between two monomers [129]. Figure 12A shows that this single-chain trimer is characterized by a tightly packed bell-shaped structure and that each individual globular domain of human adiponectin adopts a typical 10-strand jelly-roll fold, resembling the structures of members of the complement factor C1q family and the 3D-structure of proteins from the tumor necrosis factor (TNF) family [129]. It was emphasized that there is a remarkable structural and topological similarity between the globular domain of human adiponectin and tumor necrosis factor-α (TNFα), despite the fact that the amino acid sequences of these two proteins are dissimilar [127].

The adipocyte-secreted adiponectin is present in the form of trimers (∼90 kDa; the basic unit), or hexamers (∼180 kDa, also known as LMW (low-molecular-weight) form), or HMW (high-molecular-weight) species that exceed 400 kDa and ranges from 12-mers to 18-mers [127]. Trimerization of adiponectin can be prevented, and secretion of this protein can be impaired via the Arg112Cys and Ile164Thr mutations, which are clinically associated with hypoadiponectinemia [130].

It was emphasized that the full-length monomeric form of the mature adiponectin is not found under native conditions due to the thermodynamic instability of this protein [127]. However, proteolytic cleavage products containing a globular C-terminal domain were found in vivo [127]. All these facts (multifunctionality, functional regulation via PTMs, instability of monomeric form) indicate that adiponectin might possess substantial levels of intrinsic disorder. In agreement with this hypothesis, Figure 12 shows that the N-terminal half of adiponectin is predicted to be highly disordered (see Figure 12B,C) and is expected to contain multiple PTM and protein–protein interaction sites (see Figure 12C). The high binding promiscuity of human adiponectin is evidenced by both the STING-generated PPI network (see Figure 12D) and by the results of the APID-based analysis showing nine interaction partners for this protein (see Table S1).

### 2.6. Phosphoinositide 3-Kinase Regulatory Subunits 2 and 5, PIK3R2 (UniProt ID: O00459; PONDR^®^ FIT: 34.1%), and PIK3R5 (UniProt ID: Q8WYR1; PONDR^®^ FIT: 36.7%)

#### 2.6.1. Functionality of Human PI3K Proteins

Phosphatidylinositol 3,4,5-trisphosphate (PIP_3_), being one of the most important secondary messengers acting as an activator of the downstream signaling components, is known to be related to the control of cell growth, morphology, motility, proliferation, and survival by activating related signaling cascades via recruiting the PH domain-containing proteins to the membrane [131]. The PIP_3_ phospholipid is produced from phosphatidylinositol (4,5)-bisphosphate (PIP_2_) by action of the class I phosphoinositide 3-kinases (PI3-kinases, PI3Ks) responsible for phosphorylation of the PIP_2_ [132,133].

PI3Ks are heterodimers that consist of a catalytic p110 subunit (that is responsible for the kinase activity of these proteins) and a regulatory p85 subunit (that is responsible for stabilizing p110, inhibiting its catalytic activity, and recruiting PI3K to receptor or adaptor proteins) [102,134]. The complexity of PI3K proteins and their ability to initiate multiple signaling cascades that are responsible for regulating various cellular functions are determined by the fact that there are multiple isoforms of both the catalytic subunits of type 1A (p110α, p110β, and p110δ) and of type 1B (p110γ) and regulatory subunits (p85α, p85β, p55α, p50α, and p55γ) [134]. These p85-type regulatory subunits are encoded by three genes, *PIK3R1* (p85α, p55α, p50α), *PIK3R2* (p85β), and *PIK3R3* (p55γ) [135], with the products of the *PIK3R1* and *PIK3R2* genes being the predominant isoforms in the insulin-sensitive tissues [136,137,138].

It was also indicated that, due to the higher prevalence of the PI3K regulatory subunits over the catalytic subunits, the significant fraction of the regulatory subunits exists as monomers that are able to interfere with the binding of p110–p85 heterodimers to phosphorylated IRS proteins [136,139]. Binding of p85 to activated receptor tyrosine kinases or adaptor proteins, such as insulin receptor substrate-1 (IRS1) and IRS2, relieves the basal repression of p110, allowing for the activation of the type 1A catalytic subunits [140]. This is one of the mechanisms by which PI3K is critically involved in metabolic and mitogenic actions regulated by insulin [141]. It was also pointed out that PI3K may play both positive [142,143] and negative [144,145] roles in insulin secretion. PI3Kγ (p100γ) is the only representative of the type 1B PI3K. This kinase is activated by G-protein-coupled receptors [146,147] and plays important roles in inflammation, cardiac function, and tumor progression [148]. PI3Kγ/p100γ is known to be expressed in mouse and human islets [149], and knocking out this isoform in mice was shown to cause impaired glucose-stimulated insulin secretion [149,150]. Subsequent analysis revealed that the PI3Kγ/p100γ controls the size of the membrane-associated pool of secretory granules, thereby regulating β-cell Ca^2+^-dependent insulin exocytosis [151]. Activity of this type 1B PI3K is regulated by a dedicated regulatory subunit, PIK3R5 (also known as the PI3-kinase p101 subunit).

#### 2.6.2. Structural Organization and Functional Intrinsic Disorder of Human PIK3R2

**Figure 13:**
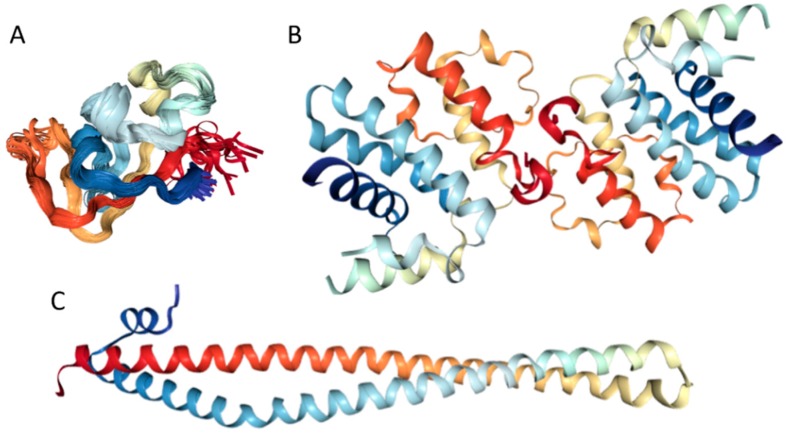
Structural characterization of human phosphoinositide 3-kinase regulatory subunits 2 (PIK3R2) protein. (**A**) Solution structure of the SH3 domain (residues 1–80, PDB ID: 2KT1). (**B**) Crystal structure of the dimeric form of the Rho-GAP domain (residues 108–298, PDB ID: 2XS6). (**C**) Crystal structure of the iSH2 domain (residues 433–610, PDB ID: 3MTT).

The p85β regulatory subunit of human PI3K (also known as PIK3R2, UniProt ID: O00459) is a 728 residue-long protein that contains one SH3 domain (residues 4–80) [152], followed by the Rho-GAP domain (residues 109–295), two Src-homology 2 (SH2) domains (residues 330–425 and 622–716) [153,154], and an iSH2 domain (the region between these two SH2 domains, residues 426–621) [155,156]. It was shown that the iSH2 domain of p85α interacts with both the ABD and C2 domains of p110α, leading to stabilization and inhibition of p110α [157,158,159,160]. Structural information is currently available for four domains of PIK3R2 (see Figure 13). 

This includes solution NMR structure of the SH3 domain (residues 1–80, PDB ID: 2KT1), crystal structure of the dimeric form of the Rho-GAP domain (residues 108–298, PDB ID: 2XS6), and crystal structure of the iSH2 domain (residues 433–610, PDB ID: 3MTT). 

**Figure 14:**
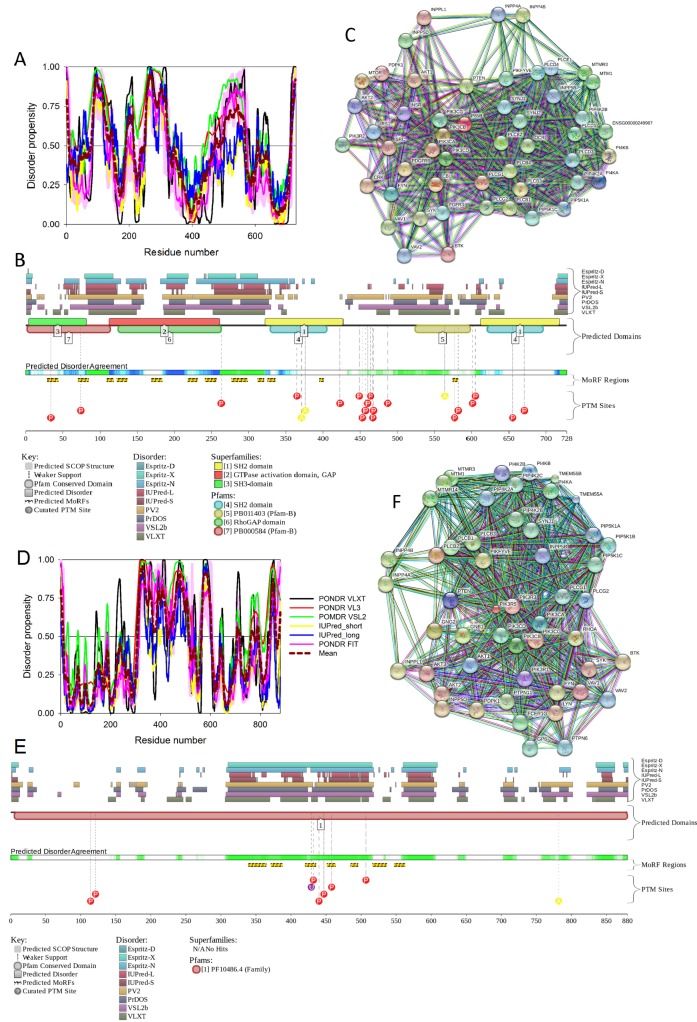
Multiparametric analysis of functional intrinsic disorder and interactability of human PIK3R2 (UniProt ID: O00459, (**A**–**C**) and PIK3R5 (UniProt ID: Q8WYR1, (**D**–**F**) proteins. (**A**,**D**) Evaluating intrinsic disorder propensity by per-residue disorder predictors, PONDR^®^ VLXT, PONDR^®^ VSL2, PONDR^®^ VL3, IUPred_short, IUPred_long and PONDR^®^ FIT. (**B**,**E**) Analysis of the intrinsic disorder propensity and some important disorder-related functional information generated by the D^2^P^2^ database (available online: http://d2p2.pro/). (**C**,**F**) STRING-based analysis of the interactivity of human PIK3R2 and PIK3R5 proteins.

Therefore, structure is known for ~66% residues of this protein. Curiously, the regions with known structure mostly coincide with the PIK3R2 regions predicted to have at least some order (see Table S1 and Figure 14A). Although no structural information is currently available for the PIK3R5 protein, data presented in Table S1 and Figure 14 indicate that both PIK3R2 and PIK3R5 are expected to have significant amounts of functional intrinsic disorder.

As shown in Figure 14A,B, the iSH2 domain of PIK3R2 is predicted to be highly disordered at residues 439–511 and 515–583; i.e., at regions that form the two major α-helices in its crystal structure [156]. Furthermore, the interhelical turn region between the α-helices (residues 512–514) exhibits the highest levels of intrinsic disorder (see Figure 14A). These observations coincide with the finding that this interhelical turn region was shown to possess a significant degree of “conformational plasticity”, which may play an important role in the function of p85 [156]. The presence of high levels of intrinsic disorder in the iSH2 domain of PIK3R2 is expected, since the structure formed by this domain is known as a coiled-coil dimer, and since coiled-coil regions in proteins are typically predicted as intrinsically disordered (and are indeed disordered, when dimers are not formed) [161,162,163,164,165,166].

The PIK3R5 regulatory subunit of human PIK3 (UniProt ID: Q8WYR1) is an 880-residue-long protein that contains a heterooligomerization region (residues 25–101) and a region needed for interaction with β-γ G protein dimers (residues 653–753). Figure 14D,E show that both of these regions are expected to be mostly ordered. However, Figure 14 shows that both PIK3R2 and PIK3R5 are predicted to contain high levels of intrinsic disorder (Figure 14A,B,D,F), have numerous sites of different PTMs (Figure 14B,E), are predicted to have multiple disorder-based interaction sites (Figure 14B,E), and are actually engaged in a broad spectrum of protein–protein interactions (Figure 14C,F). Furthermore, according to the APID server, human PIK3R2 and PIK3R5 are engaged in 148 and 8 interactions, respectively (see Table S1). Since PIK3R5 is studied to much lesser degree than PIK3R2, it is likely that the actual number of binding partners of this protein is noticeably larger than currently reported.

### 2.7. Suppressors of Cytokine Signaling, SoCS1 (UniProt ID: O15524, PONDR^®^ FIT: 34.6%) and SoCS3 (UniProt ID: O14543, PONDR^®^ FIT: 32.0%)

Suppressors of cytokine signaling (SoCS) play a number of important roles in mediating inflammatory responses in both immune and non-immune systems. SoCS proteins are promising targets for the treatment of type 2 diabetes due to their involvement in regulation of the insulin signaling and pancreatic β-cell function [167]. The SoCS family has eight members: SoCS1 to SoCS7, and cytokine-inducible SH2 domain-containing protein (CIS). They share a similar global structural organization, with a central SH2 domain followed by a SoCS box domain at their C-terminus [168]. Of the eight known members, SoCS1 and SoCS3, in conjunction with regulatory T cells, play key roles in regulation of the immune system. SoCS1 and SoCS3 contain a short motif named kinase inhibitory region (KIR) next to the SH2 domain. This KIR region is utilized in suppression of signaling by direct inhibition of catalytic activity of Janus kinases (JAKs) via binding to the JAK activation loop leading to the inhibition of kinase activity [169,170,171].

Many key proteins, particularly those associated with signaling and regulation, are known to lack stable tertiary structure yet carry out numerous biological functions [172]. The KIR regions of SoCS1 and SoCS3 have some degree of sequence identity, and the solution structure of SoCS3 has shown that KIR is unstructured [172]. 

**Figure 15:**
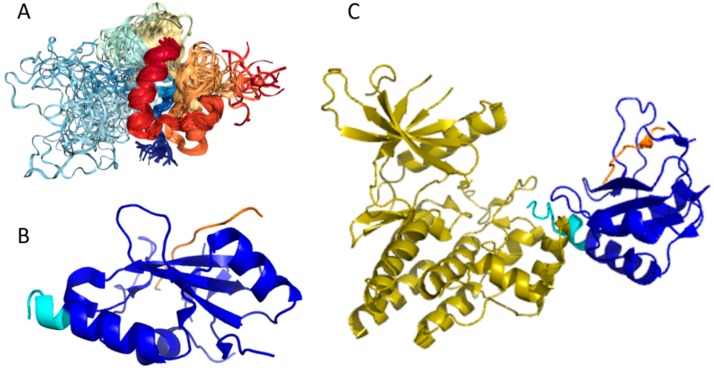
Structural characterization of murine suppressors of cytokine signaling 3 (SoCS3) protein. (**A**) Solution nuclear magnetic resonance (NMR) structure of the central region of murine SoCS3 (residues 22–161, blue-yellow structures) complexed with a phosphopeptide from the gp130 receptor (PDB ID: 2BBU, red-orange structures) [172]. (**B**) Crystal structure of the same region of murine SoCS3 protein (blue-cyan structure) complexed with gp130 (pTyr757) phosphopeptide (orange chain) (PDB ID: 2HMH) [172,173]. (**C**) Crystal structure of a ternary complex between the murine SoCS3 (residues 29–162, blue-cyan structure), protein kinase domain 2 of JAK2 (residues 835–1126, yellow structure), and a fragment of the interleuikin-6 (IL6) receptor β-chain (residues 749–763, orange chain) (PDB ID: 4GL9) [174].

Our computational analysis (provided below) revealed that the KIR regions of SoCS1 (residues 55–66) and SoCS3 (residues 22–33) are expected to have significant amounts of intrinsic disorder, which is likely to be used by KIR regions in binding to JAK [169]. Although no structural information is available for human SoCS1 and SoCS3, some interesting observations implementing roles of intrinsic disorder in protein functionality were made for the mouse SoCS3. For example, Figure 15A,B shows that the KIR region (residues 22–33) is mostly unstructured in the solution structure determined for the central region of mouse SoCS3 (residues 22–161 PDB ID: 2BBU) [172] and in the crystal structure of this protein complexed with gp130(pTyr757) phosphopeptide (PDB ID: 2HMH) [172,173]. However, Figure 15C shows that the situation becomes different when a ternary complex between the murine SoCS3 (residues 29–162), JAK2 (protein kinase domain 2, residues 835–1126), and a fragment of the IL-6 receptor β-chain (residues 749–763) is formed (PDB ID: 4GL9) [174]. Here, the SoCS3 KIR occludes the substrate-binding groove on JAK2 and blocks substrate association [174]. Therefore, although the eight residue KIR region is unstructured in isolation as shown in Figure 15A, in the structure of the ternary complex it was sharply folded back underneath the BC loop with its three N-terminal residues (Leu22–Thr24), occupying a deep groove on the JAK2 surface [174].

Another feature of the SoCS3 SH2 domain is the presence of a 35-residue unstructured loop with sequence similarity to the Pro-Glu-Ser-Thr-rich (PEST) motif [172,175]. In fact, the secondary structure assignment of mouse SoCS3 by NMR identified an insertion of 35 unstructured residues in the SH2 domain. This insertion fits the criteria for a PEST sequence but is not required for phosphotyrosine binding, suggesting that this motif has a functional role unrelated to interaction with phosphotyrosines, possibly mediating efficient proteolytic degradation of the protein [172].

The PEST motif is thought to promote proteolytic degradation, usually via the proteasome. Indeed, there is an increase in the half-life of SoCS3 when this region is deleted, indicating that degradation of this protein via the PEST motif may be an important mechanism for limiting cytokine signaling inhibition by SoCS3 [176].

**Figure 16:**
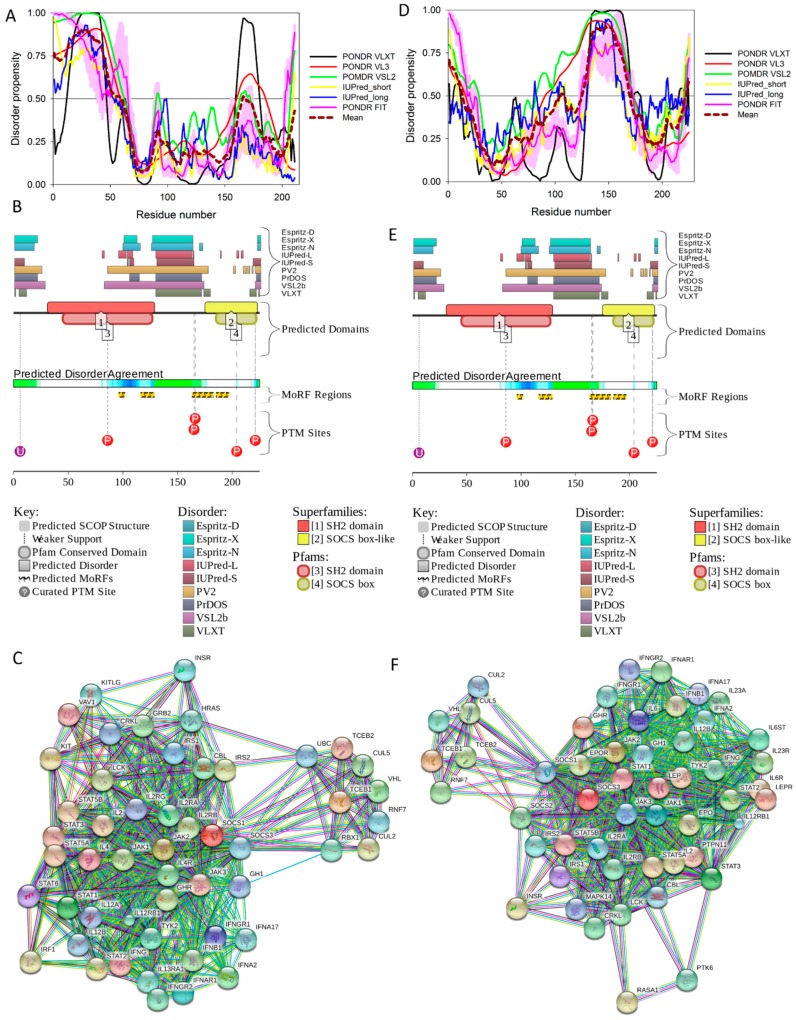
Multiparametric analysis of functional intrinsic disorder and interactability of human SoCS1 (UniProt ID: O15524, (**A**–**C**)) and SoCS3 (UniProt ID: O14543, (**D**–**F**)) proteins. (**A**,**D**) Evaluating intrinsic disorder propensity by per-residue disorder predictors, PONDR^®^ VLXT, PONDR® VSL2, PONDR^®^ VL3, IUPred_short, IUPred_long, and PONDR^®^ FIT. Plots (**B**,**E**) Analysis of the intrinsic disorder propensity and some important disorder-related functional information generated by the D^2^P^2^ database (available online: http://d2p2.pro/). (**C**,**F**) STRING-based analysis of the interactivity of human SoCS1 and SoCS3 proteins.

Figure 16 shows that the KIR regions of SoCS1 (residues 55–66) and SoCS3 (residues 22–33) are intrinsically disordered. The activation loop engaged in binding of the JAK2 is also unstructured [172]. The crystallographic studies showed that intrinsically disordered KIR regions could bind JAK2 in close proximity to the activation loop [172,175]. These studies have identified the KIR region of the SoCS1 as a promising target for the development of the SoCS1 mimetics [177]. It was also hypothesized that, due to their structural and functional independence from the remaining protein regions, the unstructured (intrinsically disordered) segments of target proteins can be used as potential therapeutics for negative and positive regulation of various biological processes, including immune response [178].

Figure 16 shows that SoCS proteins contain other disordered regions, some of which could be related to interaction with the elongin BC cullin-5 ubiquitin ligase [179]. In fact, the elongin BC complex binding domain of SoCS1 is known as BC-box characterized by the consensus sequence [APST]-L-x3-C-x3-[AILV] (residues 172–182). This BC-box motif is a part of the SoCS box (residues 161–210), which is predicted to be highly disordered, as shown in Figure 16A,B.

Also, for SoCS3, the interaction with the elongin BC complex was shown to depend on the first 12 residues of the SoCS box domain (residues 177–189) [179], which are also shown to be highly intrinsically disordered (see Figure 16D,E). These observations suggest that the elongin BC complex binding domain of SoCS proteins is expected to be disordered in isolation and only becomes structured upon elongin BC. Therefore, conformational flexibility is likely to be a key feature of the SoCS–elongin BC interaction. As far as the overall interactivity of human SoCS1 and SoCs3 is concerned, Figure 16C,F shows that both proteins serve as centers of well-developed PPI networks, and, according to APID, are respectively engaged in interactions with 71 and 86 binding partners (see Table S1).

### 2.8. Insulin (UniProt ID: P01308, PONDR^®^ FIT: 16.3%)

#### 2.8.1. Function and Structural Organization of Human Insulin

Insulin is a 51-residue-long protein hormone with the molecular mass of 5,808 Da produced by pancreatic islet β-cells. Mature insulin comprises A and B chains linked by two disulphide bridges and is possibly the most-studied protein [180] (e.g., in PubMed, there were 368,268 papers dealing with insulin as of August 17, 2017). This close attention to insulin is not surprising, since this protein is already the most commonly used therapeutic due to its link to diabetes (which is an insulin deficiency state) and the usage of this protein as a therapeutic agent is expected to further increase (up to a staggering 16,000 kg/year, if not higher) in light of the expected increase in the number of diabetic patients worldwide to ~300,000,000 by the year 2025 [181].

Insulin is an anabolic hormone known to be responsible for regulation of the metabolism of carbohydrates, fats, and proteins by promoting the absorption of small molecules (such as glucose) from the blood into liver, adipose, and skeletal muscle cells, where these small molecules are converted into large molecules, e.g., glycogen and body fat [182]. However, when insulin levels in the blood are low, widespread catabolism can be promoted. The best-known role of insulin is in the control of blood glucose levels from becoming too high (hyperglycemia) or too low (hypoglycemia) via stimulation of glucose cellular intake in muscle and adipose tissues [183]. Curiously, blood levels of insulin have profound physiological and pathological roles themselves. In fact, type 1 diabetes development is associated with the destruction of pancreatic islet β-cells by an autoimmune reaction leading to the insufficient synthesis and secretion of insulin into the blood, whereas T2DM is also characterized by the altered levels of insulin that starts forming amyloid fibrils in the pancreatic islets [184]. Furthermore, the tendency of mature insulin to form large aggregates represents significant mechanical problems in insulin delivery devices, and may cause complications during diabetes treatment.

In addition to playing a crucial role in diabetes, abnormal insulin levels may be also related to cancer pathogenesis. In fact, it is believed that hyperinsulinemia, hyperglycemia, and chronic inflammation are responsible for a direct link between diabetes and cancer [185,186,187]. Elevated levels of insulin represent a known risk factor for a number of cancers, such as colorectal, pancreatic, and breast [188]. One of the reasons for this connection is in the abundant presence on the surface of tumor cells of a short form of insulin receptor (IR-A), whose activation elicits more mitogenic than metabolic effects [189]. Therefore, insulin may favor cancer progression and facilitate the growth of tumors by stimulating this receptor [190].

Furthermore, the similarity between insulin and insulin-like growth factor (IGF) and their corresponding receptors may also have a number of effects on cancer cells. In fact, it is known that human tumors commonly over-express IR and IGF1 receptors [191], whose interaction with their corresponding ligands results in the activation of multiple signals leading to phosphorylation of adaptor proteins, such as the insulin receptor substrate family [93]. As a result, proliferation and protection from apoptotic stimuli are fostered, and promotion and progression of many types of cancer in the form of increased invasion and metastasis are enhanced [93]. Finally, it is believed that hyperinsulinemia can indirectly promote carcinogenesis through the insulin effects on IGF1 [192], since high insulin levels have been shown to stimulate IGF1 leading to increasing the risk of colorectal cancer [193]. It was also pointed out that these potential connections between insulin and cancer should be taken into account while developing modern insulin analogues to avoid generation of new oncogenic species with high binding affinity to the IGF1 receptor [194].

Insulin is a member of the insulin/IGF/relaxin superfamily that, in humans, includes a subfamily of insulin and insulin-like growth factors (IGFs) and a subfamily of relaxin peptides (such as relaxins 1, 2, and 3, Leydig cell-specific insulin-like peptide, placenta insulin-like peptide (ELIP), and insulin-like peptides 5 and 6) [195], whose members are synthesized as prepro-proteins consisting of four domains (pre, B, C, A). Maturation of the members of this superfamily includes proteolytic removal of the pre-domain. In human insulin (UniProt ID: P01308, 110 residues), the post-translational processing process is more complex and involves not only the proteolytic removal of pre-domain (or signal peptide, residues 1–24), but also proteolytic removal of the C-domain (or propeptide, residues 57–87). Such processing generates mature insulin, which is a well-folded globular protein, in which short A and B domains (residues 90–110 and 25–54, respectively) are covalently linked by two disulfide bonds (Cys31-Cys96 and Cys43-Cys109), and where the chain A is further stabilized by an additional disulfide bond (Cys95-Cys100).

**Figure 17:**
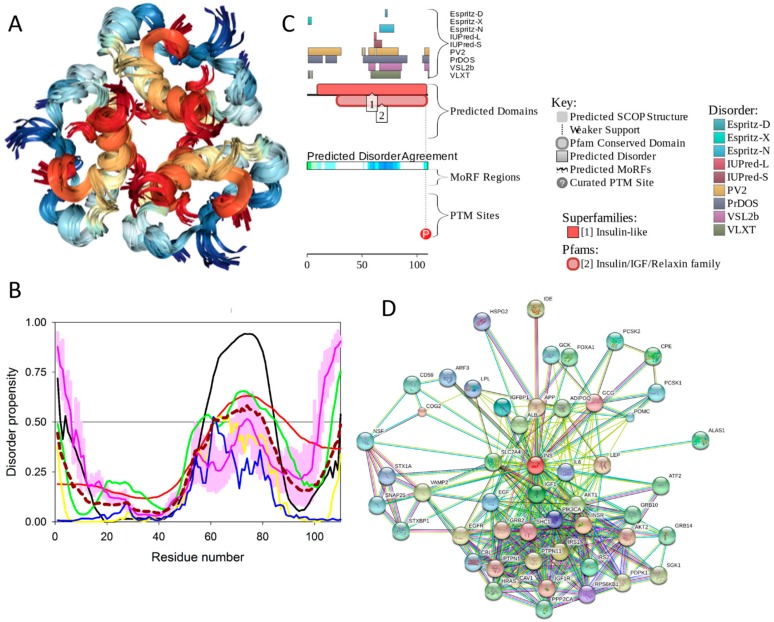
Structure and intrinsic disorder of human insulin (UniProt ID: P01308). (**A**) NMR solution structure of the insulin hexamer (PDB ID: 1AIY). This structure was generated by the NGL Viewer [201]. (**B**) Evaluating intrinsic disorder propensity of human insulin by series of per-residue disorder predictors. Disorder profiles generated by PONDR^®^ VLXT, PONDR^®^ VSL2, PONDR^®^ VL3, IUPred_short, IUPred_long, and PONDR^®^ FIT. (**C**) Analysis of the intrinsic disorder propensity and some important disorder-related functional information generated for human insulin by the D^2^P^2^ database (available online: http://d2p2.pro/). (**D**) STRING-based analysis of the interactivity of human insulin.

Physiologically, the predominant storage form of insulin is a zinc-coordinated hexamer, formed by the association of three dimers, with zinc coordination playing a crucial role in the proinsulin and insulin biogenesis [196] (see Figure 17A). In fact, the zinc-free form of insulin is a dimer at low protein concentrations over the pH 2–8 range, but it exists as a tetramer at high protein concentrations [197]. Although in in vitro solutions, insulin exists as an equilibrium mixture of monomers, dimers, tetramers, hexamers, and possibly higher associated states (depending on concentration, pH, metal ions, ionic strength, and solvent composition) [197], upon exposure to some harsh conditions—such as elevated temperatures, low pH, and organic solvents—it can also form amyloid-like fibrils [198,199].

#### 2.8.2. Prevalence and Functionality of Intrinsic Disorder of Human Insulin

To evaluate the predisposition of the pre-proinsulin for intrinsic disorder, the amino acid sequence of this protein was subjected to the multiparametric computational analysis using members of the PONDR family, such as PONDR^®^ VLXT [200], PONDR^®^ VSL2 [23], PONDR^®^ VL3 [24], and PONDR^®^ FIT [20], and also utilizing the IUPred platform [25] (see Figure 17B) In these analyses, residues/regions with predicted intrinsic disorder scores (PIDSs) above 0.5 are considered to be disordered, whereas regions with a disorder score 0.2 < PIDS < 0.5 are considered flexible. We also used the D^2^P^2^ platform to gain more information on the prevalence of intrinsic disorder in human pre-proinsulin (see Figure 17C). Figure 17B,C shows that, according to this complex analysis, several regions of the pre-proinsulin are expected to have significant amounts of intrinsic disorder. In fact, in pre-proinsulin, noticeable parts of the signal peptide (residues 1–24) and the propeptide (or the C-domain, residues 57–87) are predicted to be disordered. Therefore, sites of the proteolytic attack needed for the maturation of this protein hormone are located either within the disordered (PIDS > 0.5) or flexible (PIDS > 0.2) regions. Finally, Figure 17D shows that human insulin is engaged in a multitude of protein–protein interactions, which is in accord with the APID-based analysis showing 27 interactors for human insulin (see Table S1). It is likely that this binding promiscuity of insulin is related to a wide spectrum of biological roles assigned to this protein.

### 2.9. Insulin Receptor (UniProt ID: P06213, PONDR^®^ FIT: 14.0%)

#### 2.9.1. Functional Roles of Human Insulin Receptor

The insulin receptor (IR) is a large (1,382-residue-long) disulphide-linked plasma membrane-resident glycoprotein that forms an (αβ)_2_ homodimer [202], which is assembled from α and β chains derived from the same gene product [203], and which is heavily decorated by multiple N-linked glycans [204]. Being a tyrosine kinase receptor, IR is responsible for transmitting the extracellular signal initiated by the binding of insulin and other ligands of the insulin family into a multitude of intracellular signaling cascades, leading to profound short-term metabolic effects, as well as showing some longer term effects related to the regulation of development and growth [203,205,206,207]. IR plays a crucial role in controlling glucose homeostasis, and is thereby involved in the regulation of the metabolism of carbohydrates, lipids, and proteins [94]. In addition to being present in tissues traditionally considered as classic insulin targets, such as the adipose tissue, liver, muscle, and pancreas, insulin and the IR are also present in the brain [208,209,210]. It has been also established that, by interaction with the neuron-located IR, insulin can affect brain electrical activity, acting as a neurotransmitter or as neuromodulator and altering the release and re-uptake of other neurotransmitters, such as norepinephrine and dopamine [211,212].

IR may be expressed in two isoforms, IR-A (short or fetal isoform) and IR-B (long isoform), differing by 12 amino acids due to the alternative splicing of exon 11, with residues 745–756 missing in the IR-A isoform [213]. These isoforms have rather different biological roles, with IR-B eliciting mostly metabolic effects, and with activation of IR-A triggering more mitogenic than metabolic effects [189]. It was already pointed out that over-expression of IR is commonly observed in human tumors [191]. Importantly, IR isoforms are differently expressed by malignant cells, with expression of the IR-A isoform being predominant [189]. Although IR-A has comparable affinities to insulin and IGF2, interactions with these ligands have very different results, with binding of insulin to IR-A showing primarily metabolic effects, and IR-A activation by IGF2 leading to mitogenic effects [189]. Therefore, the aberrant IR signaling can be related not only to the T2DM pathogenesis [94], but can also be associated with Alzheimer’s disease [214] and cancer [215].

#### 2.9.2. Structural Organization and Functional Intrinsic Disorder of Human IR

IR (UniProt ID: P06213) is synthesized in the form of a precursor, with a signal peptide containing residues 1–27 removed during maturation. Furthermore, IR exists as a preformed disulfide-linked dimer, and during the proteolytic processing, the protein is cleaved at the insert domain region, generating subunits α (residues 28–758 (1–731)) and β (residues 763–1382 (738–1,355)) linked by a single intra-monomer disulfide bond Cys674-Cys899 (or Cys647-872 using numbering of the mature protein with removed signal peptide). The N-terminal extracellular part of each αβ protomer (residues 28–956 in the full-length protein or residues 1–929 in the mature protein) is known as the ectodomain. It includes the whole α-chain and 194 residues of the β-chain. The ectodomain contains two leucine-rich repeat domains, L1 (residues 1–157 using numbering of mature protein with removed signal peptide) and L2 (residues 311–475) separated by a cysteine-rich region, CR (residues 158–310); three fibronectin type-III domains, FnIII-1 (residues 476–593), FnIII-2 (594–808), and FnIII-3 (809–909); and an insert domain (ID) within FnIII-2 (residues 636–755) containing the αβ proteolytic processing site (note that the α-chain and the β-chain parts of the ID are known as an αCT segment and an IDβ region, respectively) [216].

The portion of the C-terminal cytoplasmic domain of human insulin receptor (residues 1023–1298 (981–1256)) serves as a protein tyrosine kinase (TK). The C-terminal part of the cytoplasmic domain is known as a βCT segment. This domain is linked to the remaining part of the protein via a single-pass transmembrane domain (TM, residues (909–953)) and a juxtamembrane domain (JM, residues 954–980) [217]. Therefore, in mature protein, the domain structure of subunits α and β can be described as L1–CR–L2–FNIII-1–FNIII-2–αCT and IDβ–FNIII-2–TM–JM–TM–TK–βCT, respectively. In other words, the functional α_2_β_2_ heterotetramer comprises two extracellular α subunits, whereas the two β subunits begin on the extracellular side of the membrane and then traverse the membrane into the cytoplasmic region [203].

**Figure 18:**
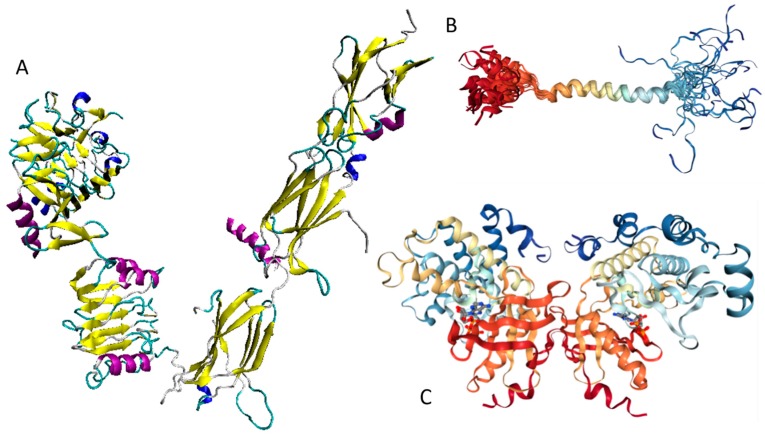
Structural characterization of different functional regions of human insulin receptor (UniProt ID: P06213). (**A**) Crystal structure of the ectodomain (residues 28–955, PDB ID: 4ZXB) [204]. (**B**) Solution NMR structure of the transmembrane domain in detergent micelles (residues 913–961, PDB ID: 2MRF) [220]. (**C**) Crystal structure of the dimeric form of the phosphorylated protein kinase domain (residues 982–1283, PDB ID: 4XLV) [221].

Despite the crucial importance of IR for understanding the molecular mechanisms of physiological and pathological actions of insulin and other ligands of the insulin family, and despite significant efforts of the scientific community, there is no structural information for the whole IR molecule due to the high flexibility of the intact IR, its membrane-bound nature, and its heavily glycosylated status [218]. However, structural information is available for several fragments of human IR, such as the N-terminal L1-CR-L2 region of ectodomain (residues 28–512, PDB ID: 2HR7) [219], the entire ectodomain (residues 28–955, PDB ID: 4ZXB, see Figure 18A) [204], the transmembrane domain in detergent micelles (residues 913–961, PDB ID: 2MRF, see Figure 18B) [220], and the protein kinase domain together and with the preceding juxtamembrane region in a phosphorylated state (residues 956–1283, PDB ID: 4XLV, see Figure 18C) [221]. Figure 18A shows that the ectodomain has a V-shaped overall structure, where the L1, CR, and L2 domains form one branch, and the FnIII-1, FnIII-2, and FnIII-3 domains constitute the other [204]. In the ectodomain structure, there are several regions of missing electron density (residues 163–176, 268–273, 516–539, 657–693, 734–753, 911–917) that are likely to correspond to IDPRs. It was also pointed out that, after Cys647 engaged in the intra-monomer α/β disulfide bridge, the major part of the ID (residues ~638–757) is largely disordered. One noticeable exception is given by the 693–710 region that forms an α-helix at binding to the surface of L1 domain on the opposing monomer [222]. Importantly, Table S1 shows that the ID region is predicted to have three MoRFs (residues 669–678 (642–651 in crystal structure), 732–743 (705–716), and 766–780 (739–753)) and that one of these MoRFs (residues 732–743 (705–716)) overlaps with the aforementioned 693–710 region, adopting an α-helical structure at the formation of a α_2_β_2_ dimer. In addition to this important observation linking intrinsic disorder with function of the IR ectodomain, flexibility and conformational dynamics of this domain were also shown to be crucial for insulin binding [217].

Figure 18B represents the NMR solution structure of the TM domain of IR (residues 913–961) in the dodecylphosphocholine (DPC) micelles [220]. Here, a well-defined α-helical structure is found for the 935–949 region, which is typical for transmembrane regions. Although the isolated JM domain (residues 954–980) that immediately follows the TM helix has not been structurally characterized yet, some structural information for this region is available from the analysis of the dimeric form of the cytoplasmic part of IR (residues 956–1,283) that, in addition to the TK domain, contains the juxtamembrane (JM) domain (see Figure 18C) [221]. 

**Figure 19:**
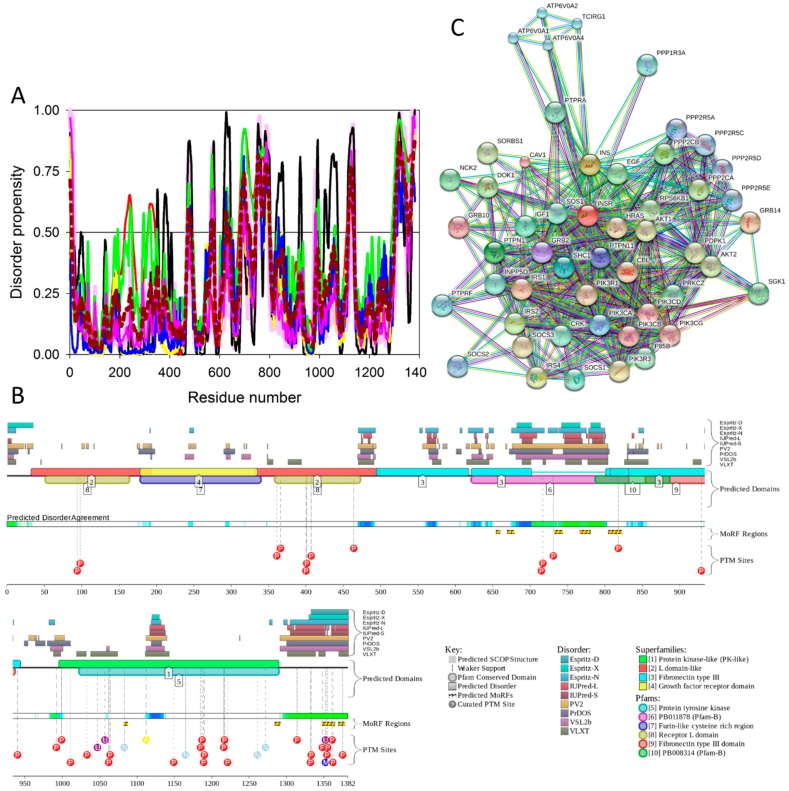
Multiparametric analysis of functional intrinsic disorder and interactability of human insulin receptor (UniProt ID: P06213). (**A**) Evaluating intrinsic disorder propensity of human insulin by series of per-residue disorder predictors. Disorder profiles generated by PONDR^®^ VLXT, PONDR^®^ VSL2, PONDR^®^ VL3, IUPred_short, IUPred_long, and PONDR^®^ FIT. (**B**) Analysis of the intrinsic disorder propensity and some important disorder-related functional information generated for human insulin receptor by the D^2^P^2^ database (available online: http://d2p2.pro/). (**C**) STRING-based analysis of the interactivity of human insulin receptor.

Analysis of this structure revealed that no electron density was found for the region corresponding to the large part of the JM domain (residues 956–973), after which a short α-helix is present. Since in the homodimeric TK domain structure, this JM-derived helix from one protomer was bound in the shallow cleft between the β-sheet and α-helix C in the N-lobe of the kinase domain from the second molecule and vice versa, it was suggested that the JM region may be involved in regulation of the TK function, acting as both as a *cis*-autoinhibitory element and a *trans*-activation element [221]. According to the multifactorial disorder analysis, the entire JM domain is expected to be mostly disordered (see Figure 19A,B), suggesting a potential role of intrinsic disorder in the function of this domain. Furthermore, both STRING- (Figure 19C) and APID-based (Table S1) analyses agreed that human IR is a promiscuous binder interacting with 136 partners (see Table S1).

Although the TK domain of the IR possesses a well-organized structure typical for the protein tyrosine kinases, with an N-terminal lobe (N-lobe) containing a five-stranded β-sheet and a single α-helix (αC), and a mainly α-helical C-terminal C-lobe that includes most of the catalytic residues spread over the catalytic and activation loops [203]. The activation loop (residues 1177–1199 (1150–1172) contains three sites of tyrosine autophosphorylation—Y1185 (1158), Y1189 (1162), and Y1190 (1163)—which are phosphorylated in *trans* following the insulin binding to the ectodomain [203]. This autophosphorylation promotes the transition of the activation loop from the autoinhibitory configuration to a conformation optimally suited for substrate binding and catalysis [203].

Overall, Figure 19 and Table S1 show that human IR contains multiple disordered regions, has at least nine MoRFs, and is heavily decorated by various PTMs. This protein is also engaged in a multitude of protein–protein interactions.

## 3. Experimental Section

### 3.1. Dataset

We investigated all 34 proteins from the KEGG-generated network of T2DM-related proteins (see Figure 1 and Table S1). Special attention was paid to ten T2DM proteins predicted to have high disorder content; i.e., proteins whose content of disordered residues predicted by PONDR^®^ FIT (CDRFIT) exceeded 30%. These proteins are: pancreatic β-cell-specific transcriptional activator, or V-MAF musculoaponeurotic fibrosarcoma oncogene homolog A, or transcription factor MAFA (UniProt ID: Q8NHW3, CDRFIT: 73.2%); insulin receptor substrates, IRS1, IRS2, and IRS4 (IRS1: UniProt ID: P35568, CDRFIT: 70.0%; IRS2: UniProt ID: Q9Y4H2, CDRFIT: 75.6%; IRS4: UniProt ID: O14654; CDRFIT: 64.4%); pancreatic and duodenal homeobox 1 protein (PDX1, UniProt ID: P52945, CDRFIT: 60.4%); phosphoinositide 3-kinase regulatory subunits 2 and 5 (PIK3R2: UniProt ID:O00459; CDRFIT: 34.1%) and PIK3R5 (UniProt ID: Q8WYR1; CDRFIT: 36.7%); suppressors of cytokine signaling SoCS1 and SoCS3 (SoCS1: UniProt ID: O15524, CDRFIT: 34.6%; and SoCS3, UniProt ID: O15524, CDRFIT: 32%); and adiponectin (UniProt ID: Q15848, CDRFIT: 38.93%). We also analyzed insulin (UniProt ID: P01308; CDRFIT: 16.3%) and insulin receptors (UniProt ID: P06213; CDRFIT: 14.03%) in more detail, due to the key role of these proteins in the T2DM pathogenesis.

### 3.2. Computational Analyses of the Amino Acid Sequences of T2DM Biomarkers

In order to analyze the residue level of disorder propensity of the T2DM related proteins, we used several computational tools for predicting the per-residue disorder propensities of a query protein (PONDR^®^ FIT [20], PONDR^®^ VLXT [21], and PONDR^®^ VSL2 algorithms [22,23,29] together with the PONDR^®^ VL3 predictor that possesses high accuracy in finding long IDPRs [24]). PONDR^®^ FIT is a consensus artificial neural network (ANN) prediction method [20], which was developed by combining the outputs of several individual disorder predictors including PONDR^®^ VLXT, PONDR^®^ VL3, PONDR^®^ VSL2, IUPred [25], FoldIndex [223], and TopIDP [224]. PONDR^®^ VLXT has significant advantages in finding potential binding sites, though it may underestimate the occurrence of long disordered regions in proteins [59,60]. The PONDR^®^ VSL2 is one of the more accurate stand-alone disorder predictors for analyzing proteins containing both ordered and disordered regions [21,225,226]. PONDR^®^ VL3 is sensitive to long IDPRs, and, is therefore better for the wholly disordered proteins. We also used the IUPred web server that allows characterization of both short and long IDPRs in query proteins [25]. For each protein, after obtaining an average disorder score by each predictor, all predictor-specific average scores were averaged again to generate an average per-protein intrinsic disorder score. The use of consensus for evaluation of intrinsic disorder is motivated by empirical observations that this approach usually increases the predictive performance compared to the use of a single predictor [225,227,228]. The outputs of all these per-residue disorder predictors are real numbers between 1 and 0, where 1 is the ideal prediction of disorder and 0 is the ideal prediction of order. The residues/regions with disorder scores above 0.5 are considered to be disordered, the residues/regions with disorder scores ranging from 0.25 to 0.5 are considered flexible, whereas the residues/regions with disorder scores below 0.25 are considered ordered.

In addition to these per-residue predictors of intrinsic disorder, we utilized binary disorder predictors that evaluate the predisposition of a query protein to be ordered or disordered as a whole. The outputs of two of these tools, the charge-hydropathy (CH) plot [30,31] and the cumulative distribution function (CDF) plot [31,229], were combined to generate the CH-CDF plot [229,230,231]. In this plot, the coordinates of a query protein are calculated as a following: the Y-coordinate corresponded to the distance of the point representing this protein in the CH-plot from the boundary (ΔCH), whereas the X-coordinate was an average distance of the respective CDF curve from the CDF boundary (ΔCDF). In the resulting CH-CDF plot, positive and negative Y-values corresponding to proteins predicted by CH-plot to be extended or compact, respectively. Positive and negative X-values correspond to proteins predicted to be ordered or intrinsically disordered by CDF analysis. The CH-CDF phase space provides specific expectations for the disorder status of a protein, depending on its position within the four quadrants. Here, the upper-right quadrant Q1 contains proteins predicted to be disordered by CH-plot but ordered by CDF; the lower-right quadrant Q2 is occupied by ordered proteins; the lower-left quadrant Q3 includes proteins that are predicted as disordered by CDF but compact by CH-plot (i.e., native molten globules or hybrid proteins containing comparable quantities of order and disorder); whereas the upper-left quadrant Q4 contains proteins with extended disorder, such as native coils and native pre-molten globules [230].

To analyze the consensus intrinsic disorder and to find disorder-based interaction sites, molecular recognition features (MoRFs), the MobiDB database [26,27], the ANCHOR algorithm [61,62], and the MoRFchibi system [232] were used. The MobiDB database combines different data sources related to protein disorder into a consensus annotation, and was used to analyze the consensus intrinsic disorder. The database incorporates data from X-ray/NMR structures and multiple intrinsic disorder predictors to evaluate the possible disorder segments of a given protein of interest [26,27]. The ANCHOR algorithm (available online: http://anchor.enzim.hu/) is used to predict protein binding regions that are disordered in isolation but can undergo disorder-to-order transition upon binding. The algorithm captures segments within disorder regions that cannot form stable intrachain interactions to fold on their own, but are likely to gain stabilizing energy by interacting with a globular protein partner [61,62].

The use of disorder predictors to find potential protein binding sites is based on the observation that the sharp dips of order within predicted disordered regions could indicate the presence of the short, loosely structured binding regions that undergo disorder-to-order transitions on interaction with the specific binding partners. MoRFs are short potentially ordered segments within longer disordered regions that bind to globular protein domains and undergo disorder-to-order transition. These disorder-based binding sites are categorized into three types: α-MoRFs (form α-helices upon binding), β-MoRFs (form β-strands), and ι-MoRFs (form irregular structures). MoRFchibi system contains three MoRFs predictors: MoRFCHiBi, a basic predictor best suited as a component in other applications; MoRFCHiBi_Light, ideal for high-throughput predictions; and MoRFCHiBi_Web, slower than the other two but best for highly accurate predictions [232]. We use a cut off-value around 0.7 with more than four residues above this cut-off identified as MoRFs.

To provide more information on the presence of functional disordered regions in T2DM related proteins, we also utilized the D^2^P^2^ internet database [49] (available online: http://d2p2.pro/), which is a community database for the pre-computed disorder predictions. D^2^P^2^ combines outputs of PONDR^®^ VLXT, IUPred, PONDR^®^ VSL2B [24,48], PrDOS [50], ESpritz [51], and PV2 [49] to show disorder predisposition of a query protein. It is further enhanced by the information on the curated sites of various posttranslational modifications and on the location of predicted disorder-based potential binding sites.

Finally, STRING (Search Tool for the Retrieval of Interacting Genes) databases were used to find the interactivity of T2DM-related proteins. They are an online resource that provide both experimental and predicted interaction information for query proteins [68].

## 4. Conclusions

This work was dedicated to the computational analysis of several proteins from the KEGG database that have been experimentally shown to be involved in T2DM. The major focus of this analysis was on T2DM-related proteins with high levels of intrinsic disorder, studied in order to better understand the potential roles of protein disorder in the pathogenesis of this disease. Our analysis systematically showed that IDPRs of the T2DM-related proteins are frequently engaged in protein–protein interactions and contain sites of various PTMs. Our analysis also reconfirmed that intrinsic disorder-associated posttranslational modifications play important roles in controlling functions of intrinsically disordered proteins. It is likely that considering T2DM proteins from the perspective of intrinsic disorder, given the known roles of IDPs/IDPRs in engaging in promiscuous interactions, can generate an important foundation for future experimental studies that may uncover latent and novel pathways associated with the disease.

## Supplementary Materials

Supplementary materials can be found at www.mdpi.com/1422-0067/18/10/2010/s1.
