# Supplementary material for: A Comprehensive Survey of the Roles of Highly Disordered Proteins in Type 2 Diabetes
Source: Int J Mol Sci. 2017 Sep 21;18(10):2010. doi: 10.3390/ijms18102010 (PMC5666700; doi:10.3390/ijms18102010)
Supplement: Supplementary file 1 [file ijms-18-02010-s001.pdf]

Supplementary Materials

A Comprehensive Survey of the Roles of Highly Disordered Proteins in Type 2 Diabetes

Zhihua Du [1,2]* and Vladimir N. Uversky[1,3,4]*

[1] *Department of Molecular Medicine, Morsani College of Medicine, University of South Florida, 12901 Bruce B. Downs Blvd. MDC07, Tampa, Florida, USA; E-mail: vuversky@health.usf.edu (V.N.U.);*

[2] *Shenzhen University, Guangdong Province, P.R. China; E-Mail: duzh@szu.edu.cn;*

[3] *USF Health Byrd Alzheimer's Research Institute, Morsani College of Medicine, University of South Florida, 12901 Bruce B. Downs Blvd. MDC07, Tampa, Florida, USA;*

[4] *Laboratory of New Methods in Biology, Institute for Biological Instrumentation, Russian Academy of Sciences, Institutskaya str., Pushchino, Moscow region 142290, Russia;*

* Authors to whom correspondence should be addressed; Z.D.: Tel: + 86-134-8063-9937; E-mail: duzh@szu.edu.cn; V. N. U.: Tel: + 01-813-974-5816. Fax: +01-813-974-7357. E-mail: vuversky@health.usf.edu

Table S1. Major disorder-related characteristics of human T2DM-related proteins.

Protein	UniProt ID	Protein length (N_{AIBS})[a]	PONDR-FIT[b]	MobiDB consensus[c]	Location (length) of long disordered regions[d]	Location (length) of AIBS[e]	N_{int}[f]	MoRF[h]
**IRS2 (Insulin receptor substrate 2)**	**Q9Y4H2**	**1338 (27/56.9)**	**75.63**	**74.81**	**1-31 (31)<br>49-72 (24)<br>303-411 (109)<br>428-537 (110)<br>840-928 (89)<br>935-1101 (167)<br>1121-1296 (176)**	**29-53 (25)<br>92-101 (10)<br>111-120 (10)<br>290-299 (10)<br>322-343 (22)<br>347-363 (17)<br>372-457 (86)<br>469-519 (51)<br>535-578 (44)<br>595-614 (20)<br>685-702 (18)<br>742-753 (12)<br>760-767 (8)<br>783-788 (6)<br>798-810 (13)<br>816-836 (21)<br>849-857 (9)<br>876-907 (32)<br>917-962 (46)<br>970-987 (18)<br>995-1018 (24)<br>1038-1087 (50)<br>1095-1125 (31)<br>1131-1196 (66)<br>1205-1262 (58)<br>1282-1304 (23)<br>1317-1338 (22)**	**58**	**31-51<br>71-84<br>571-577<br>917-924<br>1073-1076<br>1106-1110<br>1130-1138<br>1247-1259<br>1320-1338**
**MAFA (Transcription factor MAFA)**	**Q8NHW3**	**353 (8/64.6)**	**73.4**	**57.2**	**35-112 (78)<br>143-164 (22)<br>167-225 (59)<br>319-353 (35)**	**1-7 (7)<br>16-31 (16)<br>41-50 (10)<br>54-71 (18)<br>106-255 (150)<br>266-275 (10)<br>309-316 (8)<br>345-353 (9)**	**12**	**305-327<br>329-337<br>346-355<br>365-369**
**IRS1 (Insulin receptor substrate 1)**	**P35568**	**1243 (32/58.1)**	**70.7**	**72.62**	**115-158 (44)<br>268-434 (167)<br>442-460 (19)<br>494-507 (14)<br>522-695 (174)<br>704-725 (22)<br>736-755 (20)<br>765-890 (126)<br>918-984 (67)<br>1005-1018 (14)<br>1028-1154 (127)<br>1188-1242 (55)**	**26-34 (9)<br>84-89 (6)<br>107-113 (7)<br>275-289 (15)<br>295-348 (54)<br>354-393 (40)<br>403-413 (11)<br>424-432 (9)<br>436-445 (10)<br>461-472 (12)<br>478-489 (12)<br>499-529 (31)<br>539-597 (59)<br>602-676 (75)<br>688-708 (21)<br>722-736 (15)<br>759-772 (14)<br>787-806 (20)<br>813-874 (62)<br>881-886 (6)<br>894-927 (34)<br>940-965 (26)<br>971-980 (10)<br>987-1002 (16)<br>1008-1037 (30)<br>1050-1063 (14)<br>1067-1079 (13)<br>1082-1096 (15)<br>1111-1127 (17)**	**107**	**6-35<br>41-54<br>548-553<br>894-902<br>1146-1152<br>1177-1186<br>1224-1241**

						1143-1156 (14)<br>1164-1192 (29)<br>1222-1237 (16)		
PDX1 (Pancreas/ duodenum homeobox protein 1)	P52945	283<br>(8/46)	60.4	67.1	19-82 (64)<br>84-109 (26)<br>200-283 (84)	6-22 (17)<br>30-38 (9)<br>52-60 (9)<br>71-93 (23)<br>159-169 (11)<br>181-196 (16)<br>224-239 (16)<br>247-275 (29)	20	3-17<br>188-201<br>233-265
IRS4 (Insulin receptor substrate 4)	O14654	1,257<br>(22/39.1)	64.4	58.79	406-653 (248)<br>678-921 (244)<br>1179-1257 (79)	22-31 (10)<br>425-439 (15)<br>452-470 (19)<br>482-499 (18)<br>512-527 (16)<br>543-597 (55)<br>604-626 (23)<br>647-693 (47)<br>700-728 (29)<br>736-751 (16)<br>775-789 (15)<br>802-821 (20)<br>825-852 (28)<br>888-902 (15)<br>917-928 (12)<br>942-965 (24)<br>1047-1060 (14)<br>1092-1100 (9)<br>1130-1141 (12)<br>1154-1183 (30)<br>1190-1215 (26)<br>1235-1257 (23)	138	95-99<br>413-416<br>430-437<br>487-494<br>579-583<br>608-623<br>646-664<br>698-706<br>741-747<br>776-785<br>808-811<br>829-832<br>890-900<br>1018-1021<br>1155-1166<br>1218-1229<br>1236-1256
ADIPO (Adiponectin)	Q15848	244<br>(4/24.5)	38.93	30.74	16-25 (10)<br>40-101 (62)	1-15 (15)<br>26-45 (20)<br>71-81 (11)<br>109-118 (10)	9	N.P.
PIK3R5 (Phosphoinositide 3-kinase regulatory subunit 5)	Q8WYR1	880<br>(7/13.6)	36.7	29.2	312-349 (38)<br>351-370 (20)<br>386-418 (33)<br>437-515 (79)<br>563-603 (41)<br>783-793 (11)<br>838-858 (21)	339-364 (26)<br>371-387 (17)<br>420-435 (16)<br>451-463 (13)<br>485-495 (11)<br>516-536 (21)<br>547-562 (16)	8	416-434<br>555-565
SoCS1 (Suppressor of cytokine signaling 1)	O15524	210<br>(2/13.3)	34.6	25.1	1-53 (53)	1-16 (16)<br>71-81 (11)	71	1-26
PIK3R2 (Phosphatidylinositol 3-kinase regulatory subunit beta)	O00459	728<br>(12/21.42)	34.07	27.65	81-120 (40)<br>140-153 (14)<br>189-213 (25)<br>251-285 (35)<br>289-324 (36)<br>544-560 (17)<br>714-728 (15)	29-43 (15)<br>70-84 (15)<br>109-118 (10)<br>122-136 (15)<br>169-183 (15)<br>218-231 (14)<br>241-256 (16)<br>277-298 (22)<br>312-320 (9)<br>325-335 (11)<br>395-400 (6)<br>574-581 (8)	148	1-21<br>330-335
SoCS3 (Suppressor of cytokine signaling 3)	O14543	225<br>(4/22.7)	32.0	29.3	101-112 (12)<br>130-163 (34)	96-101 (6)<br>116-128 (13)<br>163-182 (20)<br>185-196 (12)	86	20-25<br>116-126<br>160-178<br>213-224
VDCC (Voltage-dependent L-type calcium channel subunit alpha-1C, CACNA1C)	Q13936	2,221<br>(20/13.8)	28.3	24.63	73-98 (26)<br>449-477 (29)<br>763-859 (97)<br>2191-2221 (31)	1-11 (11)<br>30-40 (11)<br>47-66 (20)<br>802-827 (26)<br>1743-1760 (18)<br>1768-1779 (12)<br>1795-1808 (14)	24	1-2<br>505-510<br>2101-2102<br>2207-2219

						1815-1837 (23)<br>1846-1861 (16)<br>1867-1875 (9)<br>1883-1895 (13)<br>1930-1944 (15)<br>1969-1988 (20)<br>2000-2023 (24)<br>2067-2079 (13)<br>2096-2101 (6)<br>2116-2141 (26)<br>2144-2154 (11)<br>2175-2184 (10)<br>2214-2221 (8)		
SoCS4 (Suppressor of cytokine signaling 4)	Q8WXH5	440<br>(2/3.9)	26.1	20.4	1-36 (36)<br>38-50 (13)	29-38 (10)<br>141-147 (7)	22	23-41<br>119-127<br>141-152<br>427-437
PRKCE (Protein kinase C epsilon type)	Q02156	737<br>(4/5.5)	21.4	18.1	136-163 (28)<br>310-341 (32)<br>348-364 (17)<br>373-400 (28)	299-307 (9)<br>321-327 (7)<br>355-371 (17)<br>406-413 (8)	81	38-44<br>682-687
SoCS2 (Suppressor of cytokine signaling 2)	O14508	198<br>(2/12.1)	20.7	18.7	1-31 (31)<br>136-145 (10)	1-8 (8)<br>36-51 (16)	24	1-5<br>19-22
JNK9 (Mitogen-activated protein kinase 9, MAPK9)	P45984	424<br>(3/12)	18.4	18.2	366-424 (59)	320-325 (6)<br>352-362 (11)<br>391-424 (34)	143	270-279<br>321-326<br>352-356
PRKCZ (Protein kinase C zeta type)	Q05513	592<br>(2/3.7)	18.2	13.2	1-15 (15)<br>186-205 (20)<br>213-228 (16)	21-32 (12)<br>577-586 (10)	130	554-562<br>566-585
INS (insulin)	P01308	110<br>(0/0)	16.3	28.2	55-83 (29)	N.P.[h]	27	48-50
GCK (Glucokinase)	P35557	465<br>(2/2.2)	15.7	6.9	1-12 (12)	19-23 (5)<br>125-129 (5)	14	1-7<br>273-274
JNK10 (Mitogen-activated protein kinase 10, MAPK10)	P53779	464<br>(3/11.2)	15.5	23.1	1-44 (44)<br>402-464 (63)	356-363 (8)<br>390-399 (10)<br>431-464 (34)	52	N.P.
JNK8 (Mitogen-activated protein kinase 8, MAPK8)	P45983	427<br>(5/11.7)	15.4	21.31	366-379 (14)<br>380-427 (48)	319-324 (6)<br>352-363 (12)<br>377-387 (11)<br>397-402 (6)<br>413-427 (15)	226	320-326
PYK (Protein-tyrosine kinase 2-beta)	Q14289	1,009<br>(6/6.73)	14.46	14.97	1-11 (11)<br>14-23 (10)<br>363-376 (14)<br>693-734 (42)<br>755-769 (15)<br>779-793 (15)<br>852-869 (18)	347-352 (6)<br>676-683 (8)<br>729-751 (23)<br>768-779 (12)<br>801-809 (9)<br>879-888 (10)	386	9-16
INSR (Insulin receptor)	P06213	1,382<br>(9/7.4)	14.03	14.76	1-9 (9)<br>471-481 (11)<br>565-574 (10)<br>682-709 (28)<br>742-767 (26)<br>778-797 (20)<br>1119-1131 (13)<br>1305-1382 (78)	654-659 (6)<br>669-678 (10)<br>732-743 (12)<br>766-780 (15)<br>804-822 (19)<br>1081-1087 (6)<br>1284-1201 (8)<br>1347-1365 (18)<br>1369-1377 (9)	136	737-743<br>752-756<br>1348-1381
TNF-α (Tumor necrosis factor)	P01375	233<br>(3/13.7)	12.44	18.88	1-9 (9)<br>12-26 (15)<br>79-89 (11)<br>177-185 (9)	31-52 (22)<br>132-136 (5)<br>229-233 (5)	138	1-8<br>196-197
MAPK3 (ERK1, Mitogen-activated protein kinase 3, MAPK3)	P27361	379<br>(1/1.3)	11.87	9.23	1-27 (27)	1-5 (5)	276	1-2<br>329-332
Kir6.2 or IKATP (Inward rectifier K(+) channel Kir6.2 or ATP-sensitive inward	Q14654	390<br>(0/0)	11.79	8.21	366-390 (25)	N.P.	18	7-8<br>34-37<br>365-389

rectifier potassium channel 11)								
GLUT4 (Solute carrier family 2, facilitated glucose transporter member 4)	P14672	509 (1/0.98)	9.6	9.0	1-18 (18)<br>63-72 (10)<br>494-509 (16)	26-30	51	498-507
mTOR (Serine/threonine-protein kinase mTOR)	P42345	2,549 (4/1.3)	8.9	8.0	1-18 (18)<br>910-930 (21)<br>1815-1866 (52)<br>2437-2491 (55)	19-27 (9)<br>263-272 (10)<br>298-304 (7)<br>1748-1754 (7)	175	N.P.
SUR1 (Sulfonylurea receptor 1; ATP-binding cassette sub-family C member 8)	Q09428	1,581 (2/1.1)	8.85	6.58	621-641 (21)<br>744-761 (18)<br>987-935 (53)	678-686 (9)<br>920-928 (9)	6	N.P.
MAPK1 (Mitogen-activated protein kinase 1)	P28482	360 (0/0)	8.1	5.3	1-11 (11)<br>353-360 (8)	N.P.	329	308-319<br>338-359
IKKA (Inhibitor of nuclear factor kappa-B kinase subunit alpha)	O15111	745 (2/2.6)	7.38	6.31	1-15 (15)	713-723 (11)<br>738-745 (8)	186	738-744
GLUT2 (Solute carrier family 2, facilitated glucose transporter member 2)	P11168	524 (0/0)	6.3	5.53	277-288 (12)	N.P.	6	N.P.
PRKCD (Protein kinase C delta type)	Q05655	676 (1/0.3)	5.8	6.8	297-312 (16)<br>620-630 (11)	4-5 (2)	178	1-7<br>605-613<br>620-625<br>633-639
PIK3CB (Phosphatidylinositol 4,5-bisphosphate 3-kinase catalytic subunit beta isoform)	P42338	1,070 (2/0.65)	3.36	2.06	521-529 (9)	286-286 (1)<br>400-405 (6)	53	N.P.
PIK3CA (Phosphatidylinositol 4,5-bisphosphate 3-kinase catalytic subunit alpha isoform)	P42336	1,068 (0/0)	3.00	2.06	1-7 (7)<br>534-538 (5)	479-480 (2)	101	N.P.

Table is colored to indicate disorder status of analyzed proteins. Following the accepted practice, two arbitrary cutoffs for the levels of intrinsic disorder are used to classify proteins as highly ordered (PIDR<10% blue), moderately disordered (10%≤PIDR< 30%, pink) and highly disordered (PIDR≥30%, red) (50).

[a] N_{AIBS} (A/B) represents the number of potential disorder-based binding sites identified by the ANCHOR algorithm (A) and the percentage of residues involved in disorder-based interactions (B).

[b] Content of disordered residues (i.e., residues with the disorder propensity ≥0.5) in a protein based on the PONDR-FIT disorder prediction.

[c] Content of predicted disordered residues in a protein based on the MobiDB consensus score.

[d] Information on long disordered regions (i.e., disordered regions of at least 10 residues) was obtained based on the MobiDB consensus profile.

[e]AIBSs are potential disorder-based binding sites identified by the ANCHOR algorithm.

[f] N_{int}, number of interactions as found using the Agile Protein Interactomes DataServer (http://cicblade.dep.usal.es:8080/APID/init.action).
